# Quantitative MRI-based radiomics for noninvasively predicting molecular subtypes and survival in glioma patients

**DOI:** 10.1038/s41698-021-00205-z

**Published:** 2021-07-26

**Authors:** Jing Yan, Bin Zhang, Shuaitong Zhang, Jingliang Cheng, Xianzhi Liu, Weiwei Wang, Yuhao Dong, Lu Zhang, Xiaokai Mo, Qiuying Chen, Jin Fang, Fei Wang, Jie Tian, Shuixing Zhang, Zhenyu Zhang

**Affiliations:** 1 Department of MRI, The First Affiliated Hospital of Zhengzhou University, Zhengzhou, China; 2 Department of Radiology, The First Affiliated Hospital of Jinan University, Guangzhou, Guangdong China; 3 Beijing Advanced Innovation Center for Big Data-Based Precision Medicine, Beihang University, Beijing, China; 4 CAS Key Laboratory of Molecular Imaging, Institute of Automation, Chinese Academy of Sciences, Beijing, China; 5 School of Engineering Medicine, Beihang University, Beijing, China; 6 Department of Neurosurgery, The First Affiliated Hospital of Zhengzhou University, Zhengzhou, China; 7 Department of Pathology, The First Affiliated Hospital of Zhengzhou University, Zhengzhou, China; 8 Department of Catheterization Lab, Guangdong Cardiovascular Institute, Guangdong Provincial Key Laboratory of South China Structural Heart Disease, Guangdong Provincial People’s Hospital/Guangdong Academy of Medical Sciences, Guangzhou, Guangdong China; 9 Engineering Research Center of Molecular and Neuro Imaging of Ministry of Education, School of Life Science and Technology, Xidian University, Xi’an, Shanxi China

**Keywords:** CNS cancer, Cancer models

## Abstract

Gliomas can be classified into five molecular groups based on the status of IDH mutation, 1p/19q codeletion, and TERT promoter mutation, whereas they need to be obtained by biopsy or surgery. Thus, we aimed to use MRI-based radiomics to noninvasively predict the molecular groups and assess their prognostic value. We retrospectively identified 357 patients with gliomas and extracted radiomic features from their preoperative MRI images. Single-layered radiomic signatures were generated using a single MR sequence using Bayesian-regularization neural networks. Image fusion models were built by combing the significant radiomic signatures. By separately predicting the molecular markers, the predictive molecular groups were obtained. Prognostic nomograms were developed based on the predictive molecular groups and clinicopathologic data to predict progression-free survival (PFS) and overall survival (OS). The results showed that the image fusion model incorporating radiomic signatures from contrast-enhanced T1-weighted imaging (cT1WI) and apparent diffusion coefficient (ADC) achieved an AUC of 0.884 and 0.669 for predicting IDH and TERT status, respectively. cT1WI-based radiomic signature alone yielded favorable performance in predicting 1p/19q status (AUC = 0.815). The predictive molecular groups were comparable to actual ones in predicting PFS (C-index: 0.709 vs. 0.722, *P* = 0.241) and OS (C-index: 0.703 vs. 0.751, *P* = 0.359). Subgroup analyses by grades showed similar findings. The prognostic nomograms based on grades and the predictive molecular groups yielded a C-index of 0.736 and 0.735 in predicting PFS and OS, respectively. Accordingly, MRI-based radiomics may be useful for noninvasively detecting molecular groups and predicting survival in gliomas regardless of grades.

## Introduction

Every year, ~100,000 people worldwide are diagnosed as having gliomas^1^. Gliomas are the most common primary malignant central nervous system cancer, which accounts for almost 80% of malignant brain tumors, with the highest mortality and morbidity^2^. They can be classified into lower-grade (grade II/III) and higher-grade gliomas (grade IV) based on World Health Organization (WHO) criteria^3^.

Patients with gliomas may have substantially varied survival within grades^4^. Treatment planning, response monitoring, and overall prognosis assessment for glioma patients depend heavily on the genetic and epigenetic factors in each individual tumor. The new classification announced by the WHO in 2016 recognized several new entities of glioma based on isocitrate dehydrogenase (IDH) mutation and 1p/19q codeletion in addition to the histologic grades^5^. Early evidence has confirmed that gliomas with IDH mutation and 1p/19q codeletion have better survival, whereas glioblastoma with telomerase reverse transcriptase (TERT) promoter mutation have worse survival^6^. A recent study^7^ defined five molecular groups using three genetic markers: triple-positive, mutations in both TERT and IDH, a mutation in IDH only, a mutation in TERT only, and triple-negative. The molecular groups had different overall survival (OS). Intra-tumoral genetic heterogeneity is known to exist, however, it needs to be evaluated by molecular assay following invasive biopsy or surgical resection. Histopathological assessment is invasive and has sampling errors^8^. Therefore, a noninvasive and repeatable technique is of great scientific and clinical significance to predict the molecular alternations of gliomas and assess their prognostic value, which helps to designate a proper treatment strategy.

Brain magnetic resonance imaging (MRI) can noninvasively provide more comprehensive information about tumor heterogeneity than focal tissue samples, however, such information is behind the images that beyond visual perception^9^.

Recent advances in glioma stratification depend on biological genotypes and application of deep learning and/or radiomics based predictive models using MRI biomarkers to non-invasively assess the genotypes, providing potential benefits for personalized and effective treatment plans^9^. Radiomics is an emerging field that converts medical imaging data into high-dimensional hand-crafted features using an automated data mining algorithm, such as machine learning^10,11^. By contrast, deep learning is a method to mine high-dimensional numeric information by learning relevant features (termed “deep features”) directly from images^12^. By analyzing tumor spatial and temporal heterogeneity, high-throughput hand-crafted or learned features enabled to characterize diseases for molecular diagnosis, prognosis, and treatment monitoring^13–17^. These computational techniques may exhibit prospective possibilities of overcoming limitations of tissue sampling, as it considers the complete spatial extent of the tumor. In the field of gliomas, recent reviews have shown the potential of MRI-based deep learning alone, radiomics alone, and their combination (i.e., deep learning-based radiomics) in grading, molecular subtyping, and survival prediction of patients^9,18–20^. Grading of gliomas is an essential but critical issue related to prognosis and survival. Many attempts have been made to investigate the value of multi-modal MR imaging biomarker analysis based on radiomics and deep learning classification, in the noninvasive assessment of tumor heterogeneity towards the gliomas grading with encouraging findings^21–26^.

Non-invasive tumor decoding and phenotyping of gliomas have attracted extensive attention in recent years. Dozens of studies employing multimodal MRI-based models to identify IDH mutation status, followed by 1p/19q codeletion with high accuracies of more than 85%^27–46^. To date, three pilot studies applied radiomics to discriminate TERT promotor genotype in gliomas with an accuracy of around 80%^47–49^. Nevertheless, most of the previous works focused on the prediction of single genetic alteration. Also, radiomic features extracted from tumor and edema have shown the incremental value of survival prediction in gliomas when added to clinical, pathological, and genetic profiles^50^. The combination of deep features and radiomic features may achieve improved performance for survival prediction^51,52^, however, the deep features are not easy to be interpreted by physicians.

We hypothesized that the quantitative radiomic profiles from brain MRI could represent the underlying tumor genetic information and prognostic importance. To the best of our knowledge, we firstly predicted molecular groups of gliomas based on the status of IDH mutation, 1p/19q codeletion, and TERT promoter mutation using multiparametric MRI radiomics. In addition, we assessed the association of predictive molecular groups with progression-free survival (PFS) and OS. We developed prognostic nomograms incorporating the predictive molecular groups and clinicopathologic data to individually predict the PFS and OS of grade II–IV gliomas. In addition, we also performed subgroup analyses by WHO grade to determine the performance of radiomic models in molecular subtyping and survival prediction.

## Results

### Clinical and genetic characteristics of patients

The clinical characteristics of the training and validation cohorts are summarized in Supplementary Table 2. No significant differences were observed between the two cohorts (*P* = 0.075–0.897). Among the 357 glioma cases, 111 (31.1%) were grade IV, 76 (21.3%) were grade III, and 170 (47.6%) were grade II. A total of 175 (49%) patients had tumors with peritumoral edema. 165 (46.2%) cases had the IDH mutation, 95 (26.6%) had the 1p/19q codeletion, and 185 (51.8%) had TERT promoter mutation.

### Radiomic feature extraction and selection

**Fig. 1:**
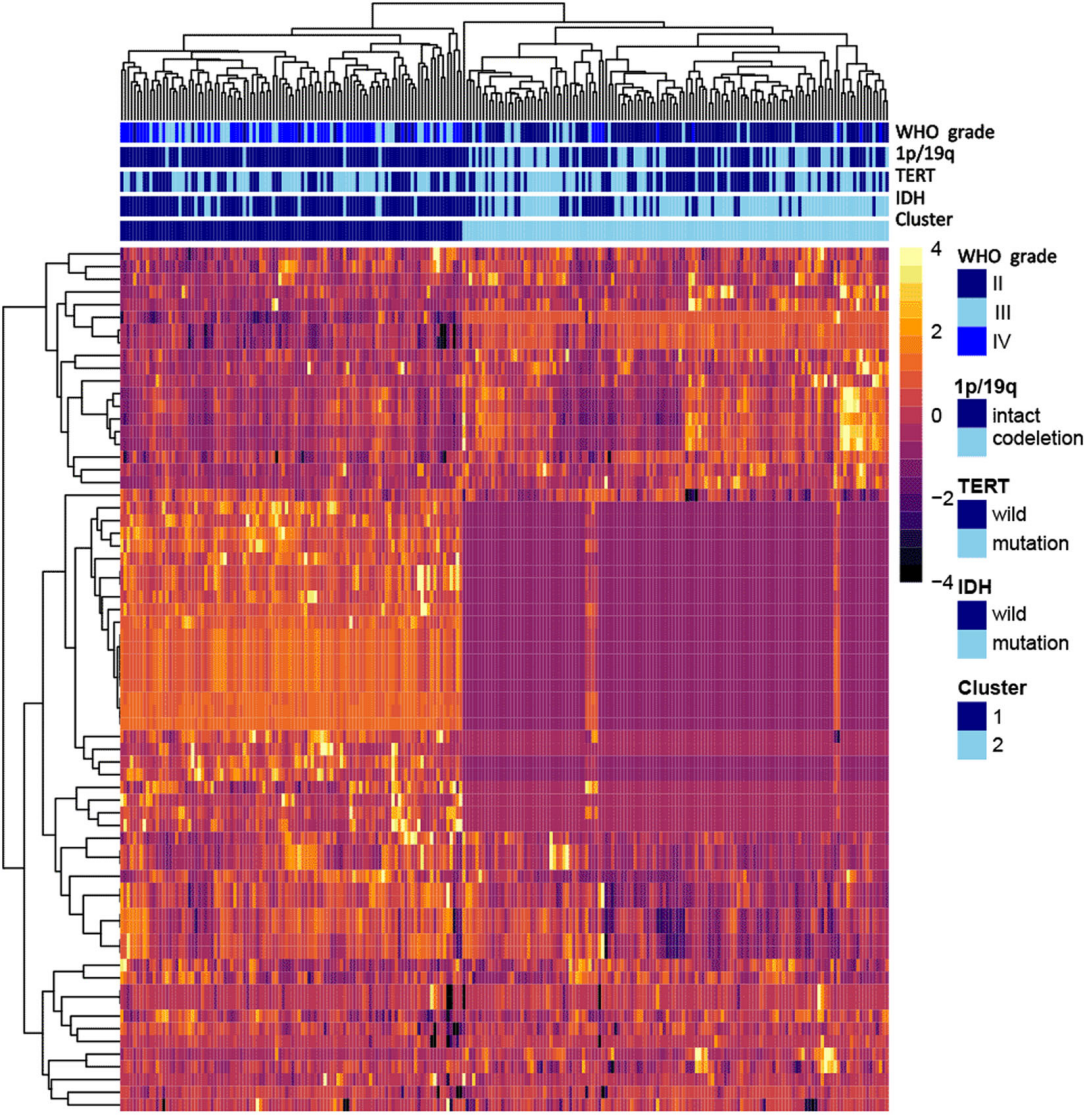
Radiomic heatmap. **a** Unsupervised clustering of patients with gliomas is shown on the *x*-axis, and radiomic features selected by LASSO for prediction of IDH mutation, 1p/19q codeletion, and TERT promoter mutation status are shown on the *y*-axis, revealing clusters of patients with similar radiomic expression patterns. **b** Correspondence of radiomic feature groups with the clustered expression patterns.

For gliomas with peritumoral edema, 8730 (=873*5*2) features were extracted from the multiparametric MRI data, whereas for gliomas without edema, 4365 (=873*5) features were extracted from the tumor region. The extracted radiomic features are available at 10.5061/dryad.j3tx95xd9. Supplementary Table 3 shows the number of retained features after each step of feature selection. More than 99% of irrelevant or highly correlated features were reduced. Supplementary Table 4 shows the final features involved in single-layered radiomic signatures for predicting IDH, 1p/19q, and TERT status. A heatmap chart with a radiomic feature dendrogram is illustrated in Fig. 1, which shows close associations between the selected MRI radiomic features and the three genetic alterations.

### Constructing image fusion models

Tables S5–7 demonstrate the performance of single-layered radiomic signatures for the prediction of IDH, 1p/19q, and TERT status, respectively. For prediction of IDH mutation status (Table 1), the image fusion model incorporating radiomic signatures based on contrast-enhanced T1-weighted imaging (cT1WI) and apparent diffusion coefficient (ADC) achieved the highest value, which was significantly superior to a clinical model based on age and tumor location (*P* < 0.001 in the training and *P* = 0.002 in the validation cohort). After adding age and tumor location to the image fusion model, no improvement was reached (*P* > 0.05). Thus, the image fusion model was used as the final model, with an area under the curve (AUC) of 0.884 (95% confidence interval [CI]: 0.830–0.934), accuracy of 0.824 (95% CI: 0.765–0.882, sensitivity of 0.750 (95% CI: 0.656–0.841), specificity of 0.898 (95% CI: 0.831–0.962), positive predictive value (PPV) of 0.882 (95% CI: 0.804–0.956), and negative predictive value (NPV) of 0.779 (95% CI: 0.693–0.857). Subgroup analysis by grades II/III versus IV showed similar accuracies. For prediction of 1p/19q codeletion status (Table 1), the cT1WI-based radiomic model achieved the best performance, which significantly outperformed the clinical model based on age and tumor location (*P* < 0.001 in the training and *P* = 0.008 in the validation cohort). The addition of age and tumor location to the cT1WI-based radiomic model showed no improvement in prediction (*P* > 0.05). Thus, the cT1WI-based model was used as the final model, with an AUC of 0.815 (95% CI: 0.751–0.878), accuracy of 0.723 (95% CI: 0.655–0.790), sensitivity of 0.794 (95% CI: 0.686–0.906), specificity of 0.694 (95% CI: 0.608–0.772), PPV of 0.509 (95% CI: 0.409–0.625), and NPV of 0.894 (95% CI: 0.828–0.955). The radiomic model in grade II/III gliomas also yielded similar accuracy. For predicting TERT promoter mutation status (Table 1), the image fusion model combing cT1WI- and ADC-based radiomic signatures achieved the best performance, with an AUC of 0.669 (95% CI: 0.580–0.748), accuracy of 0.655 (95% CI: 0.588–0.723), sensitivity of 0.841 (95% CI: 0.766–0.915), specificity of 0.446 (95% CI: 0.339–0.554), PPV of 0.631 (95% CI: 0.549–0.718), and NPV of 0.714 (95% CI: 0.585–0.833). Among the candidate clinical variables, age was the only predictor of TERT genotype, however, integration of age to the image fusion model showed no improvement in performance (*P* > 0.05). Subgroup analysis by grades II/III versus IV showed similar accuracies. Confusion matrix of the prediction of three molecular markers was provided as Supplementary Note 3. Supplementary Note 4 shows the formula of radiomic models for predicting IDH mutation, 1p19q codeletion, and TERT promoter mutation status. The prediction value for each patient, divided by training cohort and validation cohort are shown in Supplementary Fig. 3.

**Table 1:** The performance of radiomic models for predicting IDH mutation, 1p/19q codeletion, and TERT promoter mutation status.

Genotype	WHO grade	Training cohort						Validation cohort					
		AUC	ACC	SEN	SPE	PPV	NPV	AUC	ACC	SEN	SPE	PPV	NPV
IDH mutation	II–IV	0.912 (0.880–0.944)	0.870 (0.832–0.903)	0.895 (0.842–0.942)	0.850 (0.798–0.898)	0.825 (0.764–0.879)	0.911 (0.868–0.952)	0.884 (0.830–0.934)	0.824 (0.765–0.882)	0.750 (0.656–0.841)	0.898 (0.831–0.962)	0.882 (0.804–0.956)	0.779 (0.693–0.857)
	II/III	0.867 (0.814–0.914)	0.842 (0.794–0.885)	0.929 (0.885–0.969)	0.712 (0.613–0.803)	0.829 (0.767–0.888)	0.870 (0.792–0.941)	0.895 (0.831–0.948)	0.827 (0.765–0.889)	0.791 (0.683–0.886)	0.868 (0.775–0.951)	0.872 (0.784–0.952)	0.786 (0.674–0.884)
	IV	0.823 (0.743–0.894)	0.932 (0.836–0.918)	0.333 (0.182–0.500)	0.985 (0.952–1.000)	0.667 (0.500–1.000)	0.943 (0.841–0.928)	0.860 (0.778–0.929)	0.816 (0.658–0.816)	0.647 (0.556–0.741)	0.952 (0.833–1.000)	0.917 (0.889–1.000)	0.769 (0.400–0.667)
1p/19q codeletion^a^	II–IV	0.794 (0.732–0.842)	0.706 (0.655–0.752)	0.918 (0.855–0.970)	0.633 (0.570–0.691)	0.463 (0.389–0.537)	0.957 (0.920–0.984)	0.815 (0.751–0.878)	0.723 (0.655–0.790)	0.794 (0.686–0.906)	0.694 (0.608–0.772)	0.509 (0.409–0.625)	0.894 (0.828–0.955)
	II/III	0.703 (0.630–0.772)	0.612 (0.552–0.673)	0.918 (0.859–0.970)	0.433 (0.354–0.514)	0.487 (0.413–0.564)	0.900 (0.824–0.964)	0.794 (0.713–0.865)	0.679 (0.593–0.753)	0.769 (0.632–0.893)	0.636 (0.536–0.736)	0.500 (0.375–0.622)	0.854 (0.763–0.932)
TERT promoter mutation	II–IV	0.686 (0.627–0.741)	0.676 (0.626–0.723)	0.836 (0.777–0.887)	0.509 (0.433–0.586)	0.641 (0.578–0.706)	0.747 (0.663–0.825)	0.669 (0.580–0.748)	0.655 (0.588–0.723)	0.841 (0.766–0.915)	0.446 (0.339–0.554)	0.631 (0.549–0.718)	0.714 (0.585–0.833)
	II/III	0.697 (0.628–0.763)	0.673 (0.612–0.733)	0.763 (0.684–0.837)	0.588 (0.500–0.673)	0.635 (0.554–0.713)	0.725 (0.635–0.809)	0.602 (0.497–0.710)	0.654 (0.568–0.741)	0.800 (0.698–0.896)	0.472 (0.333–0.611)	0.655 (0.542–0.750)	0.654 (0.500–0.810)
	IV	0.598 (0.470–0.722)	0.685 (0.589–0.767)	0.976 (0.930–1.000)	0.290 (0.150–0.429)	0.651 (0.551–0.746)	0.900 (0.714–1.000)	0.811 (0.689–0.918)	0.658 (0.526–0.789)	0.944 (0.842–1.000)	0.400 (0.217–0.588)	0.586 (0.433–0.733)	0.889 (0.667–1.000)

Data within parentheses represent 95% confidence interval (CI).

*AUC* area under the curve, *SEN* sensitivity, *SPE* specificity, *ACC* accuracy, *PPV* positive predictive value, *NPV* negative predictive value.

^a^The performance metrics of cT1WI-based radiomic model in prediction of 1p/19q codeletion status in grade IV gliomas could not be calculated because no patients had 1p/19q codeletion.

### Prognostic performance of the model-predicted molecular groups

Supplementary Fig. 4 shows the radiomic models could stratify most patients into five molecular groups, with significantly different PFS and OS (all log-rank tests, *P* < 0.001). The prognostic performance of the predictive molecular groups was comparable to the actual molecular groups in the training cohort (PFS: C-index, 0.757 vs. 0.745, *P* = 0.946; OS: C-index: 0.759 vs. 0.764, *P* = 0.911) and validation cohort (PFS: C-index, 0.709 vs. 0.722, *P* = 0.241; OS: C-index: 0.703 vs. 0.751, *P* = 0.359) (Table 2). When stratified by WHO grade (II/III or IV), the prognostic value of model-predicted and actual molecular groups also had no significant differences (all *P* values > 0.05) (Table 2).

**Table 2:** Prognostic performance of the predictive and actual molecular groups for predicting PFS and OS.

WHO grade	Dataset	Predictive molecular groups PFS C-index (95% CI)	Actual molecular groups PFS C-index (95% CI)	*P* value	Predictive molecular groups OS C-index (95% CI)	Actual molecular groups OS C-index (95% CI)	*P* value
II–IV	Training	0.757 (0.724–0.791)	0.745 (0.709–0.781)	0.946	0.759 (0.720–0.798)	0.764 (0.730–0.800)	0.911
	Validation	0.709 (0.656–0.763)	0.722 (0.666–0.778)	0.241	0.703 (0.644–0.762)	0.751 (0.696–0.806)	0.359
II/III	Training	0.743 (0.676–0.810)	0.739 (0.678–0.799)	0.918	0.752 (0.681–0.823)	0.778 (0.722–0.835)	0.540
	Validation	0.707 (0.624–0.790)	0.638 (0.538–0.737)	0.254	0.703 (0.611–0.796)	0.715 (0.622–0.810)	0.819
IV	Training	0.530 (0.476–0.585)	0.538 (0.468–0.608)	0.879	0.534 (0.481–0.588)	0.500 (0.431–0.568)	0.296
	Validation	0.542 (0.456–0.628)	0.528 (0.430–0.626)	0.824	0.542 (0.459–0.626)	0.522 (0.422–0.621)	0.742

*WHO* World Health Organization, *PFS* progression-free survival, *OS* overall survival, *CI* confidence interval.

### Prognostic performance of the combined nomograms

**Fig. 2:**
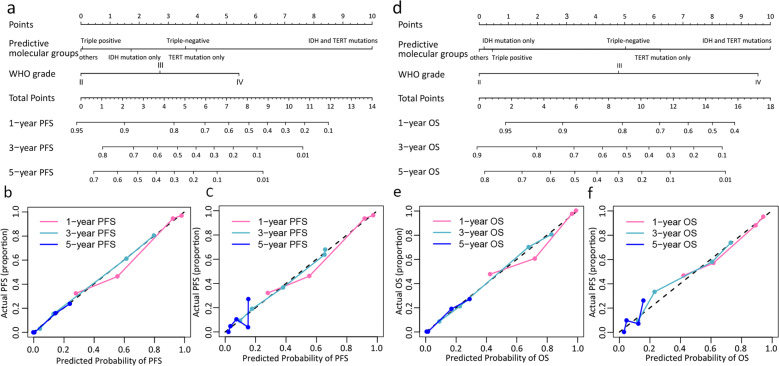
The nomograms and calibration curves. (**a**) Combined nomogram incorporating the predictive molecular groups and WHO grade for predicting PFS; (**b**-**c**) Calibration curves of the nomogram (**a**) in the training and validation datasets, respectively; (**d**) Combined nomogram incorporating the predictive molecular groups and WHO grade for predicting OS; (**e**-**f**) Calibration curves of the nomogram (**d**) in the training and validation datasets, respectively.

The prognostic nomogram for predicting PFS included WHO grade and predictive molecular groups, achieving a C-index of 0.799 (95% CI: 0.731–0.868) and 0.736 (95% CI: 0.628–0.844) in the training and validation cohorts, respectively. The prognostic nomogram for predicting OS included WHO grade and predictive molecular groups, achieving a C-index of 0.806 (95% CI: 0.740–0.872) and 0.735 (95% CI: 0.621–0.848) in the training and validation cohorts, respectively. The nomograms and calibration curves for predicting PFS and OS are shown in Fig. 2. The Hosmer–Lemeshow test yielded a nonsignificant statistic (all *P* values > 0.05 for PFS and OS), which suggested a good agreement between the prediction and actual observation. The hazard ratios (HRs) and 95%CI for WHO grade and the predictive molecular groups were shown in Supplementary Note 5.

## Discussion

We constructed MR-based radiomic models for predicting the status of IDH mutation, 1p/19q codeletion, and TERT promoter mutation prior to surgery in gliomas. These machine learning models could stratify most patients into molecular groups, with significantly different PFS and OS. The model-predicted molecular groups were comparable to the actual molecular groups in predicting PFS and OS in both grade II/III and IV gliomas. The prognostic nomograms could individually predict PFS and OS with good discrimination and calibration abilities.

Multiple studies have focused on the tasks of separating IDH mutant from IDH wildtype before surgery in gliomas utilizing multimodal MR images and associating the radiophenotypic characteristics to the mutation^27–37^. Extraction of multiple imaging features such as radiomic features and/or deep features, and pooling them into a multivariate framework may provide more predictive power than a single feature of interest. Previous studies on large and small subjects (tens to hundreds) using noninvasive MRI-based models have demonstrated that IDH genotype can be identified with mean accuracies of over 80%^27–37^. The majority of the studies to date have mainly used online open-source data, such as The Cancer Imaging Archive and The Cancer Genome Atlas. Our real-world data for the prediction of IDH status achieved high accuracy. A consensus from previous studies shows that the attributes computed from cT1WI and T2-fluid attenuated inversion recovery (T2-FLAIR) have been highly distinctive of IDH mutation than the ones computed from T1-weighted imaging (T1WI) and T2-weighted imaging (T2WI) MRI^27–37^, which were in line with our study. Ren et al.^32^ found that the histogram features on the ADC map obtained by diffusion-weighted imaging (DWI) were the most powerful factor for discriminating IDH status. However, a biological understanding of these findings remains to be elucidated. In this current study, ADC features were also a significant component of IDH status prediction. Tan et al.^30^ showed that MRI-based radiomics for the prediction of IDH status performed much better than the clinico-radiological model. Similar findings were also illustrated by our study in which age and tumor location were associated with IDH mutation but the addition of both failed to improve the accuracy of radiomic model.

Variety of radiomic features such as shape, size, histogram, texture, and wavelet have been analyzed for 1p/19q status prediction. Out of these, texture features carried a greater discriminative power when compared with other types of features^43–46^. Our study also indicated that textural features were the most crucial features for identifying 1p/19q co-deletion status. To date, the value of MRI-based radiomics for 1p/19q status prediction has not been fully explored. Our study showed that for identifying 1p/19q, feature sets derived from cT1WI had significantly higher predictive power than those from other MR sequences. Age and tumor location played a vital role in 1p/19q discrimination^53,54^ However, Han Y et al.^43^ found that integration of clinical variables into MRI-based radiomic model could not improve the prediction, which was supported by our study.

Very few studies have applied non-invasive MRI-based models to predict TERT promoter mutation is lower-grade or high-grade gliomas. Tian et al.^49^ developed a radiomic model integrating radiomic signature, age, necrotic volume percentage, Cho/Cr, and Lac to evaluate TERT status in high-grade gliomas. Tumor location was not a useful predictor for TERT status^49^. Jiang et al.^47^ concluded that MRI-based tumoral radiomic signature could evaluate TERT status in low-grade gliomas regardless of IDH status. However, the inclusion of peri-tumoral features did not improve the predictive performance^47^. Interestingly, our study observed similar findings. All radiomic features selected for identifying TERT status were tumor-related, which differed from the feature spectrum of IDH and 1p/19q status. This may partly explain why the accuracy of radiomic model for TERT was lower than the models for IDH and 1p/19q. Further studies are warranted to explore the role of MRI-based noninvasive models in delineating TERT status, for instance, deep learning.

Until now, only several studies predicted molecular subtypes of gliomas using radiomic approach. The analysis of molecular groups in gliomas will enable a more comprehensive understanding of imaging-to-molecular associations. Arita H. et al.^48^ identified three molecular subtypes (IDH-mutation, IDH-mutation with TERT promoter mutation, and IDH-wild type) in grade II/III gliomas, with an accuracy of 0.56. Similarly, Lu et al.^35^ built a three-level binary classification model to predict five molecular subtypes based on histology, IDH, and 1p/19q, achieving an accuracy of 81.8%. By discriminating the status of three tumor genetic markers, we obtained the molecular groups for individuals. The results of this study showed that the predictive molecular groups may have the potential to surrogate pathology-proven molecular groups and could serve as an independent prognostic factor for PFS and OS of gliomas. The prognostic model combing WHO grade and the predictive molecular groups yielded a favorable C-index. Considering the features of importance have been highly dependent on the grade of the tumor, we performed subgroup analyses by grade on radiomic and prognostic models. The results showed that the prognostic value of model-predicted molecular groups was comparable in both lower-grade and higher-grade gliomas. However, the predictive performance of radiomic and prognostic models was better in lower-grade than that in higher-grade gliomas.

This study also has some limitations in addition to those due to its retrospective nature. Firstly, this study was performed in a single center because TERT promoter mutation status was not detected in routine clinical practice. We tested TERT status for the purpose of research. We would like to use data of gliomas from different centers that are publicly accessible in open-source datasets to perform an external validation, but the MR sequences, genetic, and survival data were insufficient. Secondly, the inclusion of advanced MR imaging parameters in addition to the conventional modalities should be considered to construct more comprehensive functional and metabolic radiomics in the genetic characterization of glioma^55^. However, these advanced imaging techniques are not routinely used in a clinical settings but usually used for the purpose of research. Thirdly, post-operative MR images were not available in ~90% of patients. The change in the radiomic features pre- and post-operation may correlate better to the clinicopathologic data and provide additional prognostic information to the models. Fourthly, our study included images acquired from different MR systems with various acquisition parameters that may affect the reliability and reproducibility of radiomic features. Hence, we performed image data preprocessing to facilitate quantification analysis and to obtain more repeatable and comparable results. Furthermore, we carried out strict feature selection and in particular, excluded the radiomic features with significant variation among different machines and parameters. Finally, we did not separate the tumor into enhancing and necrotic regions because we included 68.9% of lower-grade gliomas.

Conclusively, our study demonstrates that three radiomic models based on pre-operative MR data for noninvasive, individualized prediction of IDH mutation, 1p/19q codeletion, and TERT promoter mutation in gliomas patients regardless of grades. Our radiomic models could successfully stratify most patients into five molecular groups, with similar prognostic performance with pathology-confirmed molecular groups. We developed prognostic nomograms that can be used in clinical settings to individually predict the PFS and OS of glioma patients. Our radiomic models can be easily integrated into the clinical setting, as it is a post-processing approach that does not require changes the current brain MR-imaging protocol and will allows clinicians to make more informed decisions for better patient care. This work may benefit the patients’ diagnosis, treatment planning, and prognosis evaluation without increasing health care expenses.

## Methods

### Patient cohort

The institutional review board in all participating centers approved this retrospective study and waived the need to obtain written consent. We identified 656 consecutive patients with newly diagnosed gliomas at the neurosurgery department between January 1, 2011 and October 1, 2016. Inclusion criteria were as follows: (a) adult patients who had a histopathological diagnosis of WHO grade II–IV gliomas; (b) patients had no history of biopsy or surgery for a brain tumor; (c) baseline multiparametric MRI inclusive of T1WI, cT1WI, T2WI, T2-FLAIR, and DWI performed prior to surgery; (d) patients were treated by surgical resection; and (e) patients had known molecular alteration status, including IDH mutation, 1p/19q codeletion, and TERT promoter mutation. Patients were excluded if (a) incomplete or absent sequences in the baseline MRI (*n* = 167); (b) inadequate MR imaging quality due to substantial motion or susceptibility artifacts (*n* = 23), or (c) patients were lost to follow-up after surgery (*n* = 109). Finally, 357 patients were included and they were randomly divided into the training cohort (*n* = 238) and validation cohort (*n* = 119) at a ratio of 2:1. Supplementary Fig. 1 illustrates the inclusion and exclusion criteria. The clinical, imaging, and histopathological data included age, sex, Karnofsky performance status (KPS) score, tumor location, tumor laterality, histologic type, WHO grade, the extent of resection, molecular markers, and treatment regimens. Formalin-fixed, paraffin-embedded tissues for IDH, 1p/19q, and TERT detection were available in these cases. Mutational hotspots in IDH1, IDH2, and the TERT promoter were detected by Sanger sequencing. Chromosome 1p/19q status was evaluated by fluorescence in situ hybridization. Detailed protocols of IDH, 1p/19q, and TERT detection have been previously described^56^. Supplementary Fig. 2 presents the representative images of identifying IDH mutation, 1p/19q codeletion, and TERT promoter mutation.

### Radiomic pipeline

**Fig. 3:**
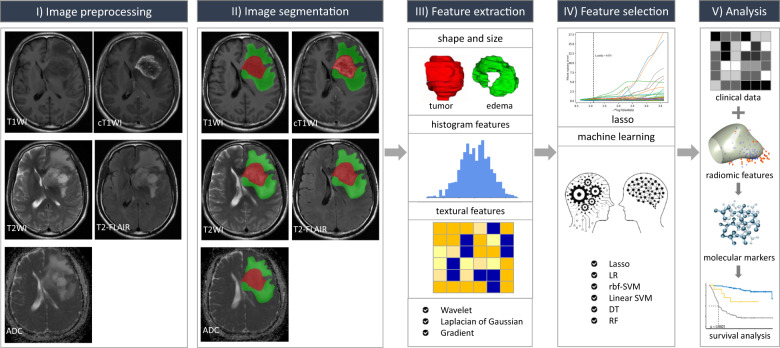
Schematic diagram of the proposed radiomic workflow for molecular subtyping and survival prediction. The study design contains five main phases: image preprocessing, image segmentation, feature extraction, feature selection, and radiomic analysis.

The radiomic process mainly comprises: (a) image pre-processing; (b) tumor and edema image segmentation; (c) feature extraction; (d) feature selection; and (e) radiomic analysis (Fig. 3).

### MR imaging and preprocessing

All patients underwent MRI examinations within one week prior to surgery. MR images were acquired in the routine clinical workup using two 1.5 T MR scanners including Achieva (Philips Medical Systems, Best, Netherlands) and Magnetom Avanto (Siemens Healthcare, Erlangen, Germany) as well as three 3.0 T MR systems, including Discovery MR750 (GE Healthcare, Milwaukee, WI, USA), Magnetom Skyra (Siemens Healthcare, Erlangen, Germany) and Magnetom Verio (Siemens Healthcare, Erlangen, Germany). The axial imaging sequences included T1WI, cT1WI, T2WI, T2-FLAIR, and DWI. ADC map was obtained by DWI (0 and 1000 s/mm^2^). The details of the MR protocol are shown in Supplementary Table 1.

Firstly, all pre-operative multimodal MR images were re-oriented to the right-anterior-inferior coordinate system using SwapDimensions function in the Functional Magnetic Resonance Imaging of the Brain (FMRIB) software library (FSL; http://fsl.fmrib.ox.ac.uk/fsl/fslwiki/FSL). Then the re-oriented MR images were registered to the re-oriented cT1WI MR images using the linear image registration tool^57,58^ with a mutual information algorithm, a Tri-Linear interpolation method, and a six degree of freedom transformation. Finally, the registered MR images were resampled to a uniform voxel size of 1 × 1 × 1 mm across all patients for radiomics construction using linear interpolation in SimpleITK (https://www.simpleitk.org).

### MR images segmentation

The three-dimensional segmentation was conducted by an open-source software ITK-SNAP (www.itk-snap.org). The region of interest (ROI) of tumor region including contrast enhancing portion (i.e., active enhancing tumor) and non-enhancing central tumor component (i.e., necrosis, if existed) was delineated on cT1WI. The edema portion was segmented using the T2-FLAIR sequence; this region was assessed based on the peritumoral hyperintensity seen on the T2-FLAIR sequence. The ROIs delineated on cT1WI and T2-FLAIR images were automatically transferred to the identical site on the T1WI, T2WI, and ADC images. The image segmentation was performed by a neuroradiologist (with 10 years of experience in neuro-radiology) and then validated by an experienced neuroradiologist (with 20 years of experience in neuro-radiology). Discrepancies between the two neuroradiologists were resolved by consensus. Neuroradiologists were blinded to the patients’ clinical and genetic information.

### Radiomic feature extraction

Prior to radiomic feature extraction, the MR images were subjected to signal intensity normalization by centering them at the mean with standard deviation (SD). Radiomic features were then extracted by using Pyradiomics 2.0.0, an open-source Python package platform (http://www.radiomics.io/pyradiomics.html). A total of 873 tumor features and 873 edema features were then extracted from T1WI, cT1WI, T2WI, T2-FLAIR, and ADC images, respectively. These features could be grouped as follows: (i) histogram-based features (*n* = 18), (ii) shape and size-based features (*n* = 13), (iii) textural features (*n* = 68), (iv) wavelet-based features (*n* = 430), (v) Laplacian of Gaussian (LoG) filter-based features (*n* = 258), and vi) features from gradient magnitude of the given MRI volumes (*n* = 86). Supplementary Note 1 provides the descriptions of image normalization and radiomic features.

### Machine learning-based single-layered radiomic signatures

High-dimensional data usually contain a majority of irrelevant, redundant, and noisy features, which could result in the curse of dimensionality and model overfitting. Therefore, feature selection should be performed to construct better generalization models when machine learning algorithms were used on high-dimensional data. Before feature selection, all features were normalized using a *z*-score approach.

A five-step feature selection process was employed by using several dimensionality reduction techniques. First step, the effect of different machine and acquisition parameters on the robustness of radiomic features was determined using the Kruskal–Wallis test, and those features that showed significant variation were excluded. Second step, *VarianceThreshold* was applied to exclude the features with low variance (threshold of 1 for IDH and 1p/19q, and threshold of 0.001 for TERT). Third step, Mann–Whitney *U* test was applied to remove features with no significant difference between the two groups (*P* ≥ 0.05). Fourth step, Pearson correction (PCC) analysis was used to assess the correlation between feature pairs and one feature was randomly excluded from each pair with a correlation coefficient > 0.9. Finally, the least absolute shrinkage and selection operator (LASSO) regression with 10-fold cross-validation was used to select the informative features with non-zero coefficients. After that, we generated five single-layered radiomic signatures based on T1WI, T2WI, T2-FLAIR, cT1WI, and ADC separately using Bayesian-regularization neural networks (BRNN). To optimize the parameters of this classifier (epoch, neuron, and mu), 10-fold cross-validation was done in the training cohort, and the optimal set of parameters for each of the classifiers was determined by the average classification performance of the classifiers in the 10 folds. The hyper-parameters of BRNN and results of 10-fold cross-validation were reported in Supplementary Note 2.

### Construction of image fusion model for predicting the molecular groups

We used multivariate logistic regression based on the stepwise bidirectional selection method to select the significant single-layered radiomic signatures and then developed image fusion models for each glioma marker prediction. Bayesian information criterion was used as the stopping rule. We also applied multivariate logistic analysis based on preoperative clinical data (age, sex, KPS score, tumor laterality, and tumor location) to build three clinical models. Predicted IDH mutation, 1p/19q codeletion, TERT promoter mutation is used to classify gliomas into five groups, mimicking the procedure to obtain the molecular groups.

### Prognostic performance of the predictive molecular groups

The primary outcomes were PFS and OS. PFS was defined as the interval between the date of surgery and either disease progression or death, censored at the last follow-up visit. Disease progression was diagnosed according to the Response Assessment in Neuro-Oncology working group criteria^59^. OS was defined as the interval from the date of initial diagnosis (date of first surgery) until the date of death, censored at the last follow-up visit. We used Kaplan–Meier survival curves with a log-rank test to compare the PFS and OS of predictive molecular groups. Also, we compared the prognostic performance (C-index) of model-predicted molecular groups with the actual molecular groups.

### Prognostic nomogram building

The candidate prognostic indicators included age, sex, KPS score, tumor location, laterality, histologic type, WHO grade, the extent of resection, radiotherapy, chemotherapy regimen, and the predictive molecular groups. The independent prognostic factors for PFS and OS were identified using a univariate and multivariate Cox regression analysis in the training cohort. Variables with *P* < 0.05 in the univariate Cox analysis that entered into multivariate Cox analysis. Those independent variables (*P* < 0.05) from the multivariate analysis were used to build a nomogram using the multivariate Cox proportional hazard model. The nomogram was independently verified in the validation cohort.

### Statistical analysis

As for continuous variables, data were expressed as mean ± SD, while for categorical variables, data were expressed as counts and percentages (n, %). Continuous and categorical variables were compared by *t* tests, Mann–Whitney *U* test, Chi-square, if appropriate. Radiomic feature extraction and selection were conducted by using Python 3.6.0 and model building was implemented by using R software (version 3.5.0). The functions within the scikit-learn package were as follows: ‘Lasso’ for LASSO, ‘brnn’ for BRNN, ‘rms’ for logistic regression analysis, Cox regression analysis, nomogram, and calibration curve, ‘ResourceSelection’ for Hosmer–Lemeshow test, ‘survminer’ for Kaplan–Meier survival curve, and ‘survival’ for C-index. To assess the association of MRI radiomic features with IDH mutation, 1p/19q codeletion, and TERT promoter mutation status, a heatmap analysis with unsupervised hierarchical clustering, one of the radiomic approaches, was performed using ‘pheatmap’ package. The AUC, accuracy, sensitivity, specificity, NPV, and PPV were calculated for prediction models and C-index was used for prognostic models. The 95% CI was obtained by 1000 stratified bootstrap replicates. The performance of prediction models was compared using the Delong test. The comparison of prognostic models using a package of ‘compared. A two-tailed *P* < 0.05 was considered statistically significant.

### Reporting summary

Further information on research design is available in the Nature Research Reporting Summary linked to this article.

## Supplementary information

Supplementary Information

Reporting Summary

## Data Availability

The data that support the findings of this study have been submitted to a generalist repositorie Dryad Digital Repository (http://datadryad.org/) and are available at 10.5061/dryad.j3tx95xd9.

## Code availability

The codes that were used to pre-process MRI images, extract radiomic features, select features, and develop the radiomic and prognostic models in the manuscript are available at https://github.com/zhang-AI-MI/glioma-genotype-radiomics.

## References

[1] Bray F (2018). Global cancer statistics 2018: GLOBOCAN estimates of incidence and mortality worldwide for 36 cancers in 185 countries. CA Cancer J. Clin..

[2] Ostrom QT (2014). The epidemiology of glioma in adults: a “state of the science” review. Neuro-Oncology.

[3] Villa C (2018). The 2016 World Health Organization classification of tumours of the central nervous system. Presse Med..

[4] Weller, M. et al. Personalized care in neuro-oncology coming of age: why we need MGMT and 1p/19q testing for malignant glioma patients in clinical practice. *Neuro-Oncology* (Suppl. 4), iv100–iv108 (2012). 10.1093/neuonc/nos206 PMC3480248 23095825

[5] Louis DN (2016). The 2016 World Health Organization Classification of tumors of the central nervous system: a summary. Acta Neuropathol..

[6] Molinaro AM (2019). Genetic and molecular epidemiology of adult diffuse glioma. Nat. Rev. Neurol..

[7] Eckel-Passow JE (2015). Glioma groups based on 1p/19q, IDH, and TERT promoter mutations in tumors. N. Engl. J. Med..

[8] Jackson RJ (2001). Limitations of stereotactic biopsy in the initial management of gliomas. Neuro-Oncology.

[9] Gore, S. et al. A review of radiomics and deep predictive modeling in glioma characterization. *Acad. Radiol.*10.1016/j.acra.2020.06.016 (2020). 10.1016/j.acra.2020.06.016 32660755

[10] Lambin P (2017). Radiomics: the bridge between medical imaging and personalized medicine. Nat. Rev. Clin. Oncol..

[11] Limkin EJ (2017). Promises and challenges for the implementation of computational medical imaging (radiomics) in oncology. Ann. Oncol..

[12] Hosny A (2018). Artificial intelligence in radiology. Nat. Rev. Cancer.

[13] Zhang B (2017). Radiomics features of multiparametric MRI as novel prognostic factors in advanced nasopharyngeal carcinoma. Clin. Cancer Res..

[14] Liu Z (2019). Radiomics of multiparametric MRI for pretreatment prediction of pathologic complete response to neoadjuvant chemotherapy in breast cancer: a Multicenter Study. Clin. Cancer Res..

[15] Trebeschi, S. et al. Predicting response to cancer immunotherapy using non-invasive radiomic biomarkers. *Ann. Oncol.*10.1093/annonc/mdz108 (2019). 10.1093/annonc/mdz108 PMC6594459 30895304

[16] Dong D (2019). Development and validation of an individualized nomogram to identify occult peritoneal metastasis in patients with advanced gastric cancer. Ann. Oncol..

[17] Kim JY (2019). Incorporating diffusion- and perfusion-weighted MRI into a radiomics model improves diagnostic performance for pseudoprogression in glioblastoma patients. Neuro-Oncol..

[18] Lohmann, P. et al. Radiomics in neuro-oncology: basics, workflow, and applications. *Methods*10.1016/j.ymeth.2020.06.003 (2020). 10.1016/j.ymeth.2020.06.003 32522530

[19] Jang K, Russo C, Di Ieva A (2020). Radiomics in gliomas: clinical implications of computational modeling and fractal-based analysis. Neuroradiology.

[20] Rudie JD (2019). Emerging applications of artificial intelligence in neuro-oncology. Radiology.

[21] Park YW (2019). Radiomics MRI phenotyping with machine learning to predict the grade of lower-grade gliomas: a study focused on nonenhancing tumors. Korean J. Radiol..

[22] Wang Q (2019). Radiomics nomogram building from multiparametric MRI to predict grade in patients with glioma: a Cohort Study. J. Magn. Reson. Imaging.

[23] Ditmer A (2018). Diagnostic accuracy of MRI texture analysis for grading gliomas. J. Neurooncol..

[24] Gutta S (2021). Improved glioma grading using deep convolutional neural networks. Am. J. Neuroradiol..

[25] Zhuge Y (2020). Automated glioma grading on conventional MRI images using deep convolutional neural networks. Med. Phys..

[26] Yang Y (2018). Glioma grading on conventional MR images: a deep learning study with transfer learning. Front. Neurosci..

[27] Choi, Y. S. et al. Fully automated hybrid approach to predict the IDH mutation status of gliomas via deep learning and radiomics. *Neuro-Oncology*10.1093/neuonc/noaa177 (2020). 10.1093/neuonc/noaa177 PMC7906063 32706862

[28] Li Z, Wang Y, Yu J, Guo Y, Cao W (2017). Deep Learning based Radiomics (DLR) and its usage in noninvasive IDH1 prediction for low grade glioma. Sci. Rep..

[29] Yu J (2017). Noninvasive IDH1 mutation estimation based on a quantitative radiomics approach for grade II glioma. Eur. Radiol..

[30] Tan Y (2019). A radiomics nomogram may improve the prediction of IDH genotype for astrocytoma before surgery. Eur. Radiol..

[31] Wu S, Meng J, Yu Q, Li P, Fu S (2019). Radiomics-based machine learning methods for isocitrate dehydrogenase genotype prediction of diffuse gliomas. J. Cancer Res. Clin..

[32] Ren Y (2019). Noninvasive prediction of IDH1 mutation and ATRX expression loss in low-grade gliomas using multiparametric MR radiomic features. J. Magn. Reson. Imaging.

[33] Peng, H. et al. Predicting isocitrate dehydrogenase (IDH) mutation status in gliomas using multiparameter MRI radiomics features. *J. Magn. Reson. Imaging*10.1002/jmri.27434 (2020). 10.1002/jmri.27434 33179832

[34] Li ZC (2018). Multiregional radiomics profiling from multiparametric MRI: Identifying an imaging predictor of IDH1 mutation status in glioblastoma. Cancer Med..

[35] Lu CF (2018). Machine learning-based radiomics for molecular subtyping of gliomas. Clin. Cancer Res..

[36] Zhang X (2018). Radiomics strategy for molecular subtype stratification of lower-grade glioma: detecting IDH and TP53 mutations based on multimodal MRI. J. Magn. Reson. Imaging.

[37] Niu L (2020). The value of enhanced MR radiomics in estimating the IDH1 genotype in high-grade gliomas. Biomed. Res. Int..

[38] Decuyper M (2020). Automated MRI based pipeline for segmentation and prediction of grade, IDH mutation and 1p19q co-deletion in glioma. Comput. Med. Imaging Graph..

[39] Yogananda CGB (2020). A novel fully automated MRI-based deep-learning method for classification of 1p/19q co-deletion status in brain gliomas. Neurooncol. Adv..

[40] Matsui Y (2020). Prediction of lower-grade glioma molecular subtypes using deep learning. J. Neurooncol..

[41] Chang P (2018). Deep-learning convolutional neural networks accurately classify genetic mutations in gliomas. Am. J. Neuroradiol..

[42] Choi KS, Choi SH, Jeong B (2019). Prediction of IDH genotype in gliomas with dynamic susceptibility contrast perfusion MR imaging using an explainable recurrent neural network. Neuro-Oncology.

[43] Han Y (2018). Non-invasive genotype prediction of chromosome 1p/19q co-deletion by development and validation of an MRI-based radiomics signature in lower-grade gliomas. J. Neurooncol..

[44] Shofty B (2018). MRI radiomics analysis of molecular alterations in low-grade gliomas. Int. J. Comput. Assist. Radiol. Surg..

[45] Kocak B (2020). Radiogenomics of lower-grade gliomas: machine learning-based MRI texture analysis for predicting 1p/19q codeletion status. Eur. Radiol..

[46] Kong Z (2020). Thin-Slice magnetic resonance imaging-based radiomics signature predicts chromosomal 1p/19q Co-deletion status in grade II and III gliomas. Front. Neurol..

[47] Jiang C (2020). Conventional magnetic resonance imaging-based radiomic signature predicts telomerase reverse transcriptase promoter mutation status in grade II and III gliomas. Neuroradiology.

[48] Arita H (2018). Lesion location implemented magnetic resonance imaging radiomics for predicting IDH and TERT promoter mutations in grade II/III gliomas. Sci. Rep..

[49] Tian H (2020). Noninvasive prediction of TERT promoter mutations in high-grade glioma by radiomics analysis based on multiparameter MRI. Biomed. Res. Int..

[50] Tan Y (2019). Improving survival prediction of high-grade glioma via machine learning techniques based on MRI radiomic, genetic and clinical risk factors. Eur. J. Radiol..

[51] Han W (2020). Deep transfer learning and radiomics feature prediction of survival of patients with high-grade gliomas. Am. J. Neuroradiol..

[52] Nie D (2019). Multi-channel 3D deep feature learning for survival time prediction of brain tumor patients using multi-modal neuroimages. Sci. Rep..

[53] Zhou H (2019). Machine learning reveals multimodal MRI patterns predictive of isocitrate dehydrogenase and 1p/19q status in diffuse low- and high-grade gliomas. J. Neurooncol..

[54] Akkus Z (2017). Predicting deletion of chromosomal arms 1p/19q in low-grade gliomas from MR images using machine intelligence. J. Digit. Imaging.

[55] Hajianfar, G. et al. Noninvasive O methylguanine-DNA methyltransferase status prediction in glioblastoma multiforme cancer using magnetic resonance imaging radiomics features: univariate and multivariate radiogenomics analysis. *World Neurosurg*. 10.1016/j.wneu.2019.08.232 (2019). 10.1016/j.wneu.2019.08.232 31505292

[56] Zhang Z (2019). Prognostic value of preoperative hematological markers combined with molecular pathology in patients with diffuse gliomas. Aging.

[57] Jenkinson M, Bannister P, Brady M, Smith S (2002). Improved optimization for the robust and accurate linear registration and motion correction of brain images. Neuroimage.

[58] Jenkinson M, Smith S (2001). A global optimisation method for robust affine registration of brain images. Med. Image Anal..

[59] Wen PY (2010). Updated response assessment criteria for high-grade gliomas: response assessment in Neuro-Oncology Working Group. J. Clin. Oncol..

