# Supplementary material for: Quantitative MRI-based radiomics for noninvasively predicting molecular subtypes and survival in glioma patients
Source: NPJ Precis Oncol. 2021 Jul 26;5:72. doi: 10.1038/s41698-021-00205-z (PMC8313682; doi:10.1038/s41698-021-00205-z)
Supplement: Supplementary file 1 — Supplementary Information [file 41698_2021_205_MOESM1_ESM.pdf]

Supplementary Materials

Supplementary Note 1: Feature extraction methods.

Supplementary Note 2: The hyper-parameters setting of Bayesian-regularization neural networks (BRNN) and the results of 10-fold cross-validation

Supplementary Note 3: Confusion matrix of the image fusion models for predicting IDH, 1p/19q, and TERT status

Supplementary Note 4: The formula of image fusion models for predicting IDH mutation, 1p19q codeletion, and TERT promoter mutation, which were built by logistic regression based on the scores of single-layered methods

Supplementary Note 5: The HRs and 95%CI for WHO grade and the predictive molecular groups in assessment of PFS and OS.

Supplementary Figure 1: Flowchart of patient inclusion.

Supplementary Figure 2: Representative results of cases in triple-positive, IDH and TERT mutations, IDH mutation only, TERT mutation only, and triple-negative groups of gliomas.

Supplementary Figure 3: The barplots for the training cohort and the validation cohort with the prediction value for each patient: (a-b) IDH prediction values in the training cohort and validation cohort; (c-d) 1p/19q prediction values in the training and validation cohorts; and (e-f) TERT prediction values in the training and validation cohorts.

Supplementary Figure 4. Kaplan-Meier curves of the predictive molecular groups. (a) PFS in the training cohort; (b) PFS in the validation cohort; (c) OS in the training cohort; and (d) OS in the validation cohort.

Supplementary Table 1: MR imaging acquisition parameters.

Supplementary Table 2: Comparison of patient and tumor characteristics between the training and validation cohort.

Supplementary Table 3: The number of retained features after each step of feature selection

Supplementary Table 4: The selected features from each MR sequence

Supplementary Table 5. The performance of single-layered radiomic signatures for prediction of IDH mutation status

Supplementary Table 6. The performance of single-layered radiomic signatures for prediction of 1p/19q codeletion status

Supplementary Table 7. The performance of single-layered radiomic signatures for prediction of TERT promoter mutation status.

Supplementary Figure 1: Flowchart of patient inclusion

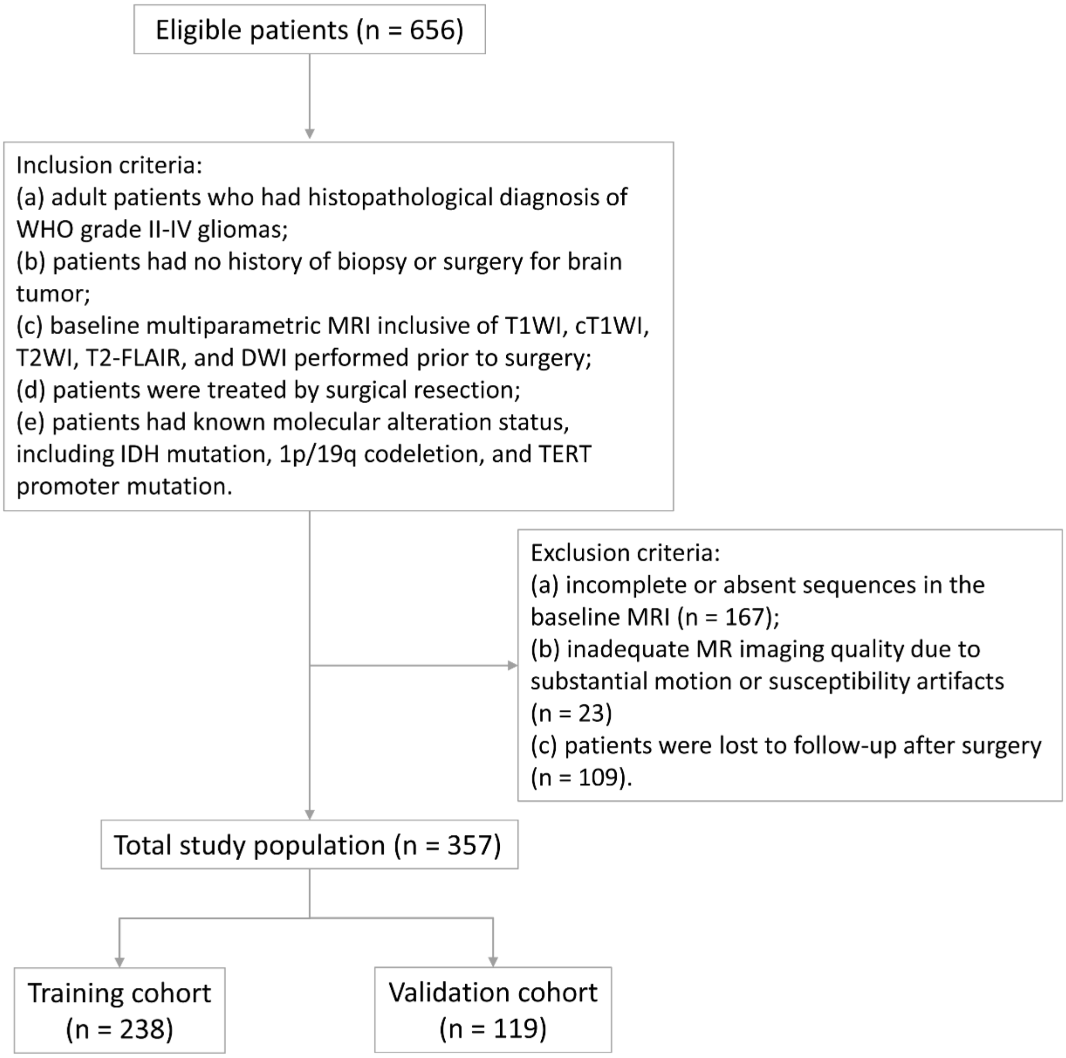

Supplementary Figure 2: Representative results of cases in triple-positive, IDH and TERT mutations, IDH mutation only, TERT mutation only, and triple-negative groups of gliomas.

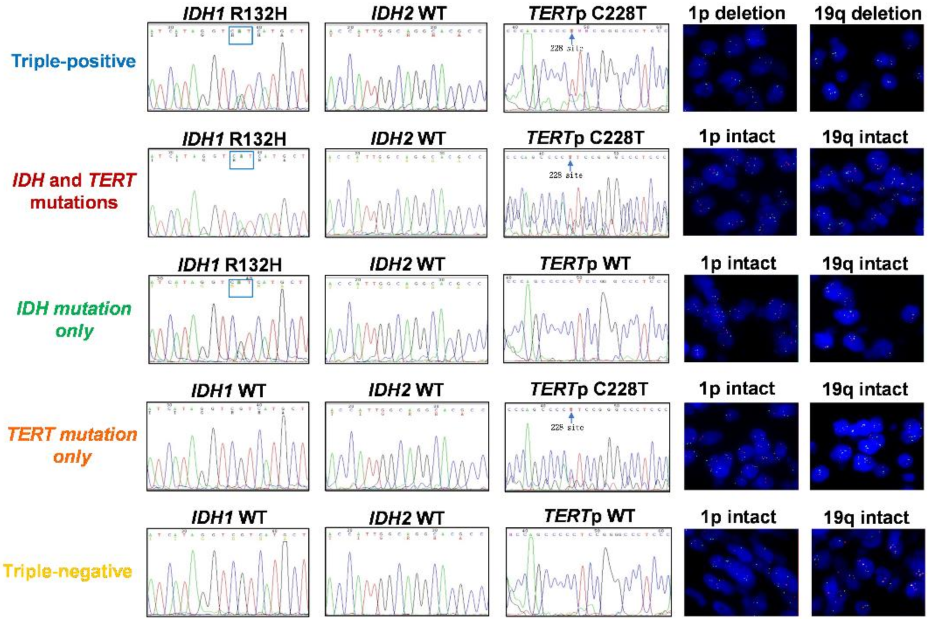

Supplementary Table 1: MR imaging acquisition parameters

All MR images were acquired on either 1.5 T or 3.0 T clinical MR scanners. The 1.5 T scanners included Siemens Healthcare (Magnetom Avanto, Erlangen, Germany) with a 12-channel head coil, and Philips Healthcare (Achieva, Best, Netherlands) with a 16-channel head coil. The 3.0 T scanners included GE Healthcare (Discovery MR750, Milwaukee, WI, USA) with an 8-channel head coil, or Siemens Healthcare (Magnetom Skyra/verio, Erlangen, Germany) with an integrated 20-channel head and neck coil and a 12-channel head coil.

Imaging parameters	Skyra (Siemens)	Verio (Siemens)	Discovery MR750 (GE)	Avanto (Siemens)	Achieva (Philips)
Field strength (T)	3	3	3	1.5	1.5
Head coil channel	20	12	8	12	16
T1WI/cT1WI					
TR (ms)	220	220	1750	202	498
TE (ms)	2.46	2.48	24	4.76	15
Section thickness (mm)	5	5	5	5	6
Intersection gap (mm)	1.5	1.5	1.5	1.5	1

FA (°)	70	80	111	70	69
NEX	1	1	1	1	1
FOV (mm)	230 × 230	220 × 206	240 × 180	230 × 187	230 × 184
Matrix	288 × 216	320 × 210	320 × 256	256 × 256	184 × 184
T2WI					
TR (ms)	5000	3900	5390	4200	2000
TE (ms)	117	92	79	90	100
Section thickness (mm)	5	5	5	5	6
Intersection gap (mm)	1.5	1.5	1.5	1.5	1
FA (°)	90	150	142	150	90
NEX	1	1	1	1	2
FOV (mm)	240 × 240	240 × 193	240 × 240	230 × 184	230 × 184
Matrix	320 × 320	320 × 210	512 × 512	320 × 256	220 × 220
T2-FLAIR					
TR (ms)	6500	6000	8400	4900	6000
TE (ms)	85	94	150	90	120
TI (ms)	2126.8	2030	2100	1780	2000
Section thickness (mm)	5	5	5	5	6
Intersection gap (mm)	1.5	1.5	1.5	1.5	1
FA (°)	150	150	111	150	90
NEX	1	1	1	1	2
FOV (mm)	240 × 240	220 × 193	240 × 240	230 × 190	230 × 198
Matrix	256 × 179	256 × 157	256 × 256	256 × 256	256 × 256
DWI					
TR (ms)	4600	3700	6000	3400	2226
TE (ms)	80	102	87	112	51
Section thickness (mm)	5	5	5	5	6
Intersection gap (mm)	1.5	1.5	1.5	1.5	1
B value (s/mm^2)	0, 1000	0, 1000	0, 1000	0, 1000	0, 1000
FA (°)	90	90	90	90	90
NEX	1	1	1	2	1
FOV (mm)	240 × 240	220 × 220	240 × 240	230 × 230	230 × 230
Matrix	192 × 192	162 × 130	160 × 160	152 × 152	112 × 112

Supplementary Note 1: Feature extraction methods

In this study, MR images were normalized before feature extraction by centering it at the mean with standard deviation in Pyradiomics 2.0.0 (http://www.radiomics.io/pyradiomics.html). The formula of normalization was shown as following:

$$f(x) = \frac{s(x - \mu_x)}{\sigma_x}$$

Where:

x and $f(x)$ represent the original and normalized MR images, respectively.

μ_x and σ_x represent the mean and standard deviation of the image intensity values, respectively.

s is a scaling factor. In this study, s was set to be 100.

After image normalization, a total of 8730 features were extracted based on the regions of tumor and edema from T1, cT1, T2, FLAIR, and apparent diffusion coefficient (ADC) map MR images. The same 873 features were extracted on both regions of tumor and edema from each sequence of MR images. These features could be divided into five groups: (I) histogram-based features (n=18), (II) shape and size-based features (n=13), (III) textural features (n=68), (IV) wavelet filter-based features (n=430), (V) Laplacian of Gaussian (LoG) filter-based features (n=258), and (VI) gradient filter features (n=86). The extraction methods of radiomic features are shown below:

❖Histogram-based features

Histogram-based features describe the distribution of pixel intensity. Let X denote the image matrix with N voxels and P the histogram with N_l discrete intensity levels. The following histogram-based features were extracted:

1. Energy:

$$energy = \sum_{i}^{N} X(i)^2$$

2. Total energy

$$total\ energy = PixelSpacing(X) \sum_{i}^{N} X(i)^2$$

3. Entropy:

$$entropy = -\sum_{i=1}^{N_l} P(i) \log_2 P(i)$$

4. Kurtosis:

$$kurtosis = \frac{\frac{1}{N}\sum_{i=1}^{N}(X(i)-\bar{X})^4}{\left(\sqrt{\frac{1}{N}\sum_{i=1}^{N}(X(i)-\bar{X})^2}\right)^2}$$

where $\bar{X}$ is the mean of X.

5. Maximum:

The maximum intensity value of X.

6. Mean:

$$mean = \frac{1}{N}\sum_{i}^{N} X(i)$$

7. Mean absolute deviation:

The mean of the absolute deviations of all voxel intensities around the mean intensity value.

8. Median:

The median intensity value of X.

9. Minimum:

The minimum intensity value of X.

10. Range:

The range of intensity values of X.

11. Root mean square (RMS):

$$RMS = \sqrt{\frac{\sum_{i}^{N} X(i)^2}{N}}$$

12. Skewness:

$$skewness = \frac{\frac{1}{N}\sum_{i=1}^{N}(X(i)-\bar{X})^3}{\left(\sqrt{\frac{1}{N}\sum_{i=1}^{N}(X(i)-\bar{X})^2}\right)^3}$$

where $\bar{X}$ is the mean of X.

13. Interquartile range:

The difference between 75th and 25th percentiles of intensity values of X.

14. 10th Percentile

The 10th percentile of intensity values of X

15. 90th Percentile

The 90th percentile of intensity values of X

16. Robust mean absolute deviation

Robust mean absolute deviation is the mean distance of all intensity values from the mean value calculated on the subset of image array with gray levels in between, or equal to the 10th and 90th percentile.

17. Uniformity:

$$uniformity = \sum_{i=1}^{N_l} P(i)^2$$

18. Variance:

$$variance = \frac{1}{N-1}\sum_{i=1}^{N}(X(i)-\bar{X})^2$$

where $\bar{X}$ is the mean of X.

❖ **Shape and size features**

This group features describe the shape and size of the tumor region. Let V represents the volume and A the surface area of the volume of interest. The following shape and size features were extracted:

1. Maximum 3D diameter

Maximum 3D diameter is defined as the largest pairwise Euclidean distance between

ROI surface mesh vertices.

2. Maximum 2D diameter (row):

   The maximum two-dimensional tumor diameter is measured as the largest pairwise Euclidean distance between voxels on the surface of the tumor volume in the column-slice plane.

3. Maximum 2D diameter (column):

   The maximum two-dimensional tumor diameter is measured as the largest pairwise Euclidean distance between voxels on the surface of the tumor volume in the row-slice plane.

4. Maximum 2D diameter (slice):

The maximum two-dimensional tumor diameter is measured as the largest pairwise Euclidean distance between voxels on the surface of the tumor volume in the row-column plane.

5. Sphericity:

$$sphericity = \frac{\pi^{\frac{1}{3}}(6V)^{\frac{2}{3}}}{A}$$

6. Surface area:

   The surface area is calculated by triangulation (i.e. dividing the surface into connected triangles) and is defined as:

$$A = \sum_{i=1}^{N} \frac{1}{2} |a_i b_i \times a_i c_i|$$

   Where N is the total number of triangles covering the surface and a, b and c are edge vectors of the triangles.

7. Surface to volume ratio:

$$surface\ to\ volume\ ratio = \frac{A}{V}$$

8. Volume:

   The volume (V) of the tumor is determined by counting the number of pixels in the tumor region and multiplying this value by the voxel size.

9. Major axis

$$major\ axis = 4\sqrt{\lambda_{major}}$$

where λ_{major} is the length of the largest principal component axis.

10. Minor axis

$$minor\ axis = 4\sqrt{\lambda_{minor}}$$

where λ_{minor} is the length of the second largest principal component axis.

11. Least axis

$$least\ axis = 4\sqrt{\lambda_{least}}$$

Where λ_{least} is the length of the least principal component axis.

12. Elongation

$$elongation = \sqrt{\frac{\lambda_{minor}}{\lambda_{major}}}$$

13. Flatness

$$flatness = \sqrt{\frac{\lambda_{least}}{\lambda_{major}}}$$

❖**Textural features**

The textural features provide the information regarding the relative position of the various gray levels over the image, and describe patterns or the spatial distribution of voxel intensities. In this study, the textural features were extracted based on four textural matrices: Gray Level Co-occurrence Matrix (GLCM), Gray Level Run Length Matrix (GLRLM), Gray Level Size Zone Matrix (GLSZM), and Gray Level Dependence Matrix (GLDM). Determining textural matrix representations requires the image intensity values within value of interest to be discretized. Image intensities were therefore resampled into equally spaced bins using a bin-width of 10 (The bin-width is determined by the grayscale range/bins of the image. In FBS, when the number of bins is set to 32, the feature is more stable. In this study, the number of bins is also set to 32. The highest point of the histogram of the grayscale distribution after image preprocessing corresponds to the grayscale range of 300, thus we set the bin-width to 10). This discretization step not only reduces image noise, but also normalizes intensities across all patients, allowing for a direct comparison of all calculated textural features between patients.

Gray Level Co-occurrence Matrix based features

A GLCM is defined as $\mathbf{P}(i,j,\delta,\alpha)$, a matrix with size $N_g \times N_g$ describing the second-order joint probability function of an image, where the (i,j)th element represents the number of times the combination of intensity levels i and j occur in two pixels in the image, that are separated by a distance δ of pixels in direction α, and N_g is the number of discrete gray level intensities. In this study, distance δ was 1 and direction α was belonging to {0°, 45°, 90°, 135°}, yielding a total of 4 GLCM for each image. From these gray level co-occurrence matrices, several textural features are derived. Each gray level co-occurrence-based feature was then calculated as the mean of the feature calculations for each of the 4 directions.

Let:

$\mathbf{P}(i,j)$ be the GLCM for an arbitrary δ and α,

N_g be the number of discrete intensity levels in the image,

μ be the mean of $\mathbf{P}(i,j)$,

$p_{\boldsymbol{x}}(i) = \sum_{j=1}^{N_g} \mathbf{P}(i,j)$ be the marginal row probabilities,

$p_{\boldsymbol{y}}(i) = \sum_{i=1}^{N_g} \mathbf{P}(i,j)$ be the marginal column probabilities,

μ_x be the mean of $p_{\boldsymbol{x}}$,

μ_y be the mean of $p_{\boldsymbol{y}}$,

σ_x be the standard deviation of $p_{\boldsymbol{x}}$,

σ_y be the standard deviation of $p_{\boldsymbol{y}}$,

$p_{x+y}(k) = \sum_{i=1}^{N_g} \sum_{j=1}^{N_g} \boldsymbol{P}(i,j), i+j=k, k=2,3,\cdots,2N_g$,

$p_{x-y}(k) = \sum_{i=1}^{N_g} \sum_{j=1}^{N_g} \boldsymbol{P}(i,j), |i-j|=k, k=0,12,3,\cdots,N_g-1$,

$HX = -\sum_{i=1}^{N_g} p_{\boldsymbol{x}}(i) log_2[p_{\boldsymbol{x}}(i)]$ be the entropy of $p_{\boldsymbol{x}}$,

$HY = -\sum_{i=1}^{N_g} p_{\boldsymbol{y}}(i) log_2[p_{\boldsymbol{y}}(i)]$ be the entropy of $p_{\boldsymbol{y}}$,

$H = -\sum_{i=1}^{N_g}\sum_{j=1}^{N_g}\boldsymbol{P}(i,j)log_2[\boldsymbol{P}(i,j)]$ be the entropy of $\mathbf{P}(i,j)$,

$HXY1 = -\sum_{i=1}^{N_g}\sum_{j=1}^{N_g}\boldsymbol{P}(i,j)log_2[p_{\boldsymbol{x}}(i)p_{\boldsymbol{y}}(i)]$,

$HXY2 = -\sum_{i=1}^{N_g}\sum_{j=1}^{N_g}p_x(i)p_y(i)log_2[p_{\boldsymbol{x}}(i)p_{\boldsymbol{y}}(i)]$,

1. Joint average

$$joint\ average = \sum_{i=1}^{N_g}\sum_{j=1}^{N_g} i\mathbf{P}(i,j)$$

2. Joint entropy

$$joint\ entropy = -\sum_{i=1}^{N_g}\sum_{j=1}^{N_g}\mathbf{P}(i,j)log_2\mathbf{P}(i,j)$$

3. Joint energy

$$joint\ energy = \sum_{i=1}^{N_g}\sum_{j=1}^{N_g}(\mathbf{P}(i,j))^2$$

4. Autocorrelation:

$$autocorrelation = \sum_{i=1}^{N_g}\sum_{j=1}^{N_g} ij\mathbf{P}(i,j)$$

5. Cluster Prominence:

$$cluster\ prominence = \sum_{i=1}^{N_g}\sum_{j=1}^{N_g}\left[i + j - \mu_x(i) - \mu_y(j)\right]^4\mathbf{P}(i,j)$$

6. Cluster Shade:

$$cluster\ shade = \sum_{i=1}^{N_g}\sum_{j=1}^{N_g}\left[i + j - \mu_x(i) - \mu_y(j)\right]^3\mathbf{P}(i,j)$$

7. Cluster Tendency:

$$cluster\ tendency = \sum_{i=1}^{N_g}\sum_{j=1}^{N_g}\left[i + j - \mu_x(i) - \mu_y(j)\right]^2\mathbf{P}(i,j)$$

8. Contrast:

$$contrast = \sum_{i=1}^{N_g}\sum_{j=1}^{N_g}|i-j|^2\mathbf{P}(i,j)$$

9. Correlation:

$$correlation = \frac{\sum_{i=1}^{N_g}\sum_{j=1}^{N_g} ij\mathbf{P}(i,j) - \mu_x(i)\mu_y(j)}{\sigma_x(i)\sigma_y(j)}$$

10. Difference entropy:

$$difference\ entropy = -\sum_{i=1}^{N_g-1} \mathbf{P}_{x-y}(i)log_2[\mathbf{P}_{x-y}(i)]$$

11. Inverse difference:

$$inverse\ difference = \sum_{i=1}^{N_g}\sum_{j=1}^{N_g}\frac{\mathbf{P}(i,j)}{1+|\boldsymbol{i}-\boldsymbol{j}|}$$

12. Informational measure of correlation 1 (IMC1):

$$IMC1 = \frac{HXY - HXY1}{\max\{HX, HY\}}$$

13. Informational measure of correlation 2 (IMC2):

$$IMC2 = \sqrt{1 - e^{1-(HXY2-HXY)}}$$

14. Inverse Difference Moment (IDM):

$$IDM = \sum_{i=1}^{N_g}\sum_{j=1}^{N_g}\frac{\mathbf{P}(i,j)}{1+|i-j|^2}$$

15. Inverse Difference Moment Normalized (IDMN):

$$IDMN = \sum_{i=1}^{N_g}\sum_{j=1}^{N_g}\frac{\mathbf{P}(i,j)}{1+\frac{|\boldsymbol{i}-\boldsymbol{j}|^2}{\boldsymbol{N}^2}}$$

16. Inverse Difference Normalized (IDN):

$$IDN = \sum_{i=1}^{N_g}\sum_{j=1}^{N_g}\frac{\mathbf{P}(i,j)}{1+\frac{|\boldsymbol{i}-\boldsymbol{j}|}{\boldsymbol{N}}}$$

17. Inverse variance:

$$inverse\ variance = \sum_{i=1}^{N_g}\sum_{j=1}^{N_g}\frac{\mathbf{P}(i,j)}{|i-j|^{\mathbf{2}}}, i \neq j$$

18. Difference average (DA):

$$difference\ average = \sum_{k=0}^{N_g-1} kp_{x-y}(k)$$

19. Difference variance (DV):

$$difference\ variance = \sum_{k=0}^{N_g-1} (k - DA)^2 p_{x-y}(k)$$

20. Maximum probability:

$$maxmium\ probability = \max\{\mathbf{P}(i,j)\}$$

21. Sum entropy:

$$sum\ entropy = -\sum_{i=2}^{2N_g} \mathbf{P}_{x+y}(i) log_2[\mathbf{P}_{x+y}(i)]$$

22. Sum squares:

$$sum\ squares = \sum_{i=1}^{N_g} \sum_{j=1}^{N_g} (i - \mu_x)^2 \mathbf{P}(i,j)$$

Gray Level Run Length Matrix based features

Run length metrics quantify gray level runs in an image. A gray level run is defined as the length in number of pixels, of consecutive pixels that have the same gray level value. In a GLRLM $p(i,j|\theta)$, the (i,j)th element describes the number of times j a gray level i appears consecutively in the direction specified by θ, and N_g is the number of discrete gray level intensities. In this study, a GLRLM was computed for each direction in set {0°, 45°, 90°, 135°}, from which the below textural features were derived. Each GLRLM based feature was then calculated as the mean of the feature values for each of the 4 directions.

Let:

$p(i,j|\theta)$ be the (i,j)th entry in the given run-length matrix p for a direction θ,

N_g the number of discrete intensity values in the image,

N_r the number of different run lengths,

N_p the number of voxels in the image.

1. Short run emphasis (SRE):

$$SRE = \frac{\sum_{i=1}^{N_g} \sum_{j=1}^{N_r} [\frac{p(i,j|\theta)}{j^2}]}{\sum_{i=1}^{N_g} \sum_{j=1}^{N_r} p(i,j|\theta)}$$

2. Long run emphasis (LRE):

$$LRE = \frac{\sum_{i=1}^{N_g}\sum_{j=1}^{N_r} j^2 p(i,j|\theta)}{\sum_{i=1}^{N_g}\sum_{j=1}^{N_r} p(i,j|\theta)}$$

3. Gray level non-uniformity (GLN):

$$GLN = \frac{\sum_{i=1}^{N_g}[\sum_{j=1}^{N_r} p(i,j|\theta)]^2}{\sum_{i=1}^{N_g}\sum_{j=1}^{N_r} p(i,j|\theta)}$$

4. Gray level non-uniformity normalized (GLNN):

$$GLNN = \frac{\sum_{i=1}^{N_g}[\sum_{j=1}^{N_r} p(i,j|\theta)]^2}{(\sum_{i=1}^{N_g}\sum_{j=1}^{N_r} p(i,j|\theta))^2}$$

5. Run length non-uniformity (RLN):

$$RLN = \frac{\sum_{i=1}^{N_r}[\sum_{j=1}^{N_g} p(i,j|\theta)]^2}{\sum_{i=1}^{N_g}\sum_{j=1}^{N_r} p(i,j|\theta)}$$

6. Run length non-uniformity normalized (RLNN):

$$RLNN = \frac{\sum_{i=1}^{N_r}[\sum_{j=1}^{N_g} p(i,j|\theta)]^2}{(\sum_{i=1}^{N_g}\sum_{j=1}^{N_r} p(i,j|\theta))^2}$$

7. Run percentage (RP):

$$RP = \sum_{i=1}^{N_g}\sum_{j=1}^{N_r}\frac{p(i,j|\theta)}{N_p}$$

8. Gray level variance (GLV):

$$GLV = \sum_{i=1}^{N_g}\sum_{j=1}^{N_r} p(i,j|\theta)(i-\mu)^2$$

Here, $\mu = \sum_{i=1}^{N_g}\sum_{j=1}^{N_r} p(i,j|\theta)i$

9. Run variance

$$RV = \sum_{i=1}^{N_g}\sum_{j=1}^{N_r} p(i,j|\theta)(j-\mu)^2$$

Here, $\mu = \sum_{i=1}^{N_g}\sum_{j=1}^{N_r} p(i,j|\theta)j$

10. Run entropy (RE):

$$RE = -\sum_{i=1}^{N_g}\sum_{j=1}^{N_r} p(i,j|\theta)log_2 p(i,j|\theta)$$

11. Low gray level run emphasis (LGLRE):

$$LGLRE = \frac{\sum_{i=1}^{N_g}\sum_{j=1}^{N_r}[\frac{p(i,j|\theta)}{i^2}]}{\sum_{i=1}^{N_g}\sum_{j=1}^{N_r}p(i,j|\theta)}$$

12. High gray level run emphasis (HGLRE):

$$HGLRE = \frac{\sum_{i=1}^{N_g}\sum_{j=1}^{N_r}i^2p(i,j|\theta)}{\sum_{i=1}^{N_g}\sum_{j=1}^{N_r}p(i,j|\theta)}$$

13. Short run low gray level emphasis (SRLGLE):

$$SRLGLE = \frac{\sum_{i=1}^{N_g}\sum_{j=1}^{N_r}[\frac{p(i,j|\theta)}{i^2j^2}]}{\sum_{i=1}^{N_g}\sum_{j=1}^{N_r}p(i,j|\theta)}$$

14. Short run high gray level emphasis (SRHGLE):

$$SRHGLE = \frac{\sum_{i=1}^{N_g}\sum_{j=1}^{N_r}[\frac{p(i,j|\theta)}{j^2}]}{\sum_{i=1}^{N_g}\sum_{j=1}^{N_r}p(i,j|\theta)}$$

15. Long run low gray level emphasis (LRLGLE):

$$LRLGLE = \frac{\sum_{i=1}^{N_g}\sum_{j=1}^{N_r}[\frac{p(i,j|\theta)j^2}{i^2}]}{\sum_{i=1}^{N_g}\sum_{j=1}^{N_r}p(i,j|\theta)}$$

16. Long run high gray level emphasis (LRHGLE):

$$LRHGLE = \frac{\sum_{i=1}^{N_g}\sum_{j=1}^{N_r}[p(i,j|\theta)j^2i^2]}{\sum_{i=1}^{N_g}\sum_{j=1}^{N_r}p(i,j|\theta)}$$

Gray level size zone matrix-based features

Gray level size zone matrices quantify gray level zones in an image. A gray level zone is defined as the number of connected voxels that share the same gray level intensity. A voxel is considered connected if the distance is 1 according to the infinity norm (26-connected region in a 3D, 8-connected region in a 2D). In a gray level size zone matrix **P**(i,j) the (i,j)th element equals the numbers of zones with gray level i and size j appear in image. The GLSZM is rotation independent, with only one matrix calculated for all directions in the ROI.

Let:

N_g be the number of discreet intensity values in the image,

N_s be the number of discreet zone sizes in the image,

N_p be the number of voxels in the image,

N_z be the number of zones in the ROI, which is equal to $\sum_{i=1}^{N_g}\sum_{j=1}^{N_s}\mathbf{P}(i,j)$ and $1 \le N_z \le N_p$,

$\mathbf{P}(i,j)$ be the size zone matrix,

$p(i,j)$ be the normalized size zone matrix, defined as $p(i,j) = \frac{\mathbf{P}(i,j)}{N_z}$

1. Small area emphasis (SAE):

$$SAE = \frac{\sum_{i=1}^{N_g}\sum_{j=1}^{N_s}[\frac{\mathbf{P}(i,j)}{j^2}]}{N_z}$$

2. Large area emphasis (LAE):

$$LAE = \frac{\sum_{i=1}^{N_g}\sum_{j=1}^{N_s}j^2\mathbf{P}(i,j)}{N_z}$$

3. Gray level non-uniformity (GLN):

$$GLN = \frac{\sum_{i=1}^{N_g}[\sum_{j=1}^{N_s}\mathbf{P}(i,j)]^2}{N_z}$$

4. Gray level non-uniformity normalized (GLNN):

$$GLNN = \frac{\sum_{i=1}^{N_g}[\sum_{j=1}^{N_s}p(i,j)]^2}{(N_z)^2}$$

5. Size zone non-uniformity (SZN):

$$SZN = \frac{\sum_{i=1}^{N_s}[\sum_{j=1}^{N_g}\mathbf{P}(i,j)]^2}{N_z}$$

6. Size zone non-uniformity normalized (RLNN):

$$SZNN = \frac{\sum_{i=1}^{N_s}[\sum_{j=1}^{N_g}\mathbf{P}(i,j)]^2}{(N_z)^2}$$

7. Zone percentage (ZP):

$$ZP = \frac{N_z}{N_p}$$

8. Gray level variance (GLV):

$$GLV = \sum_{i=1}^{N_g}\sum_{j=1}^{N_s}p(i,j)(i-\mu)^2$$

Here, $\mu = \sum_{i=1}^{N_g}\sum_{j=1}^{N_s}p(i,j)i$

9. Zone variance (ZV):

$$ZV = \sum_{i=1}^{N_g}\sum_{j=1}^{N_s} p(i,j)(j-\mu)^2$$

Here, $\mu = \sum_{i=1}^{N_g}\sum_{j=1}^{N_s} p(i,j)j$

10. Zone entropy (ZE):

$$ZE = -\sum_{i=1}^{N_g}\sum_{j=1}^{N_s} p(i,j)log_2 p(i,j)$$

11. Low gray level zone emphasis (LGLZE):

$$LGLZE = \frac{\sum_{i=1}^{N_g}\sum_{j=1}^{N_s}[\frac{p(i,j)}{i^2}]}{N_z}$$

12. High gray level zone emphasis (HGLZE):

$$HGLZE = \frac{\sum_{i=1}^{N_g}\sum_{j=1}^{N_s} i^2\mathbf{P}(i,j)}{N_z}$$

13. Small area low gray level emphasis (SALGLE):

$$SALGLE = \frac{\sum_{i=1}^{N_g}\sum_{j=1}^{N_s}[\frac{p(i,j)}{i^2 j^2}]}{N_z}$$

14. Small area high gray level emphasis (SAHGLE):

$$SRHGLE = \frac{\sum_{i=1}^{N_g}\sum_{j=1}^{N_s}[\frac{p(i,j)i^2}{j^2}]}{N_z}$$

15. Large area low gray level emphasis (LALGLE):

$$LALGLE = \frac{\sum_{i=1}^{N_g}\sum_{j=1}^{N_s}[\frac{p(i,j)j^2}{i^2}]}{N_z}$$

16. Large area high gray level emphasis (LAHGLE):

$$LAHGLE = \frac{\sum_{i=1}^{N_g}\sum_{j=1}^{N_s}[p(i,j)j^2 i^2]}{N_z}$$

Gray level dependence matrix-based features

Gray level dependence matrices quantify gray level dependencies in an image. A gray level dependency is defined as the number of connected voxels within distance δ that are dependent on the center voxel. A neighboring voxel with gray level j is considered dependent on center voxel with gray level i if |i−j|≤α. In a gray level dependence matrix **P**(i,j) the (i,j)th

element describes the number of times a voxel with gray level i with j dependent voxels in its neighborhood appears in image.

Let:

N_g be the number of discreet intensity values in the image,

N_d be the number of discreet dependency sizes in the image,

N_z be the number of dependency zones in the image, which is equal to $\sum_{i=1}^{N_g}\sum_{j=1}^{N_d}\mathbf{P}(i,j)$,

$\mathbf{P}(i,j)$ be the dependency matrix,

$p(i,j)$ be the normalized size zone matrix, defined as $p(i,j) = \frac{\mathbf{P}(i,j)}{N_z}$

1. Small dependence emphasis (SDE):

$$SDE = \frac{\sum_{i=1}^{N_g}\sum_{j=1}^{N_d}[\frac{\mathbf{P}(i,j)}{i^2}]}{N_z}$$

2. Large dependence emphasis (LDE):

$$LDE = \frac{\sum_{i=1}^{N_g}\sum_{j=1}^{N_d} j^2\mathbf{P}(i,j)}{N_z}$$

3. Gray level non-uniformity (GLN):

$$GLN = \frac{\sum_{i=1}^{N_g}[\sum_{j=1}^{N_d}\mathbf{P}(i,j)]^2}{N_z}$$

4. Dependence non-uniformity (DN):

$$DN = \frac{\sum_{i=1}^{N_d}[\sum_{j=1}^{N_g}\mathbf{P}(i,j)]^2}{(N_z)^2}$$

5. Dependence non-uniformity normalized (DNN):

$$DNN = \frac{\sum_{i=1}^{N_d}[\sum_{j=1}^{N_g}\mathbf{P}(i,j)]^2}{(N_z)^2}$$

6. Gray level variance (GLV):

$$GLV = \sum_{i=1}^{N_g}\sum_{j=1}^{N_d} p(i,j)(i-\mu)^2$$

Here, $\mu = \sum_{i=1}^{N_g}\sum_{j=1}^{N_d} p(i,j)i$

7. Dependence variance (DV):

$$DV = \sum_{i=1}^{N_g} \sum_{j=1}^{N_d} p(i,j)(j-\mu)^2$$

Here, $\mu = \sum_{i=1}^{N_g} \sum_{j=1}^{N_d} p(i,j)j$

8. Dependence entropy (DE):

$$DE = -\sum_{i=1}^{N_g} \sum_{j=1}^{N_d} p(i,j) log_2 p(i,j)$$

9. Low gray level emphasis (LGLE):

$$LGLE = \frac{\sum_{i=1}^{N_g} \sum_{j=1}^{N_d} [\frac{p(i,j)}{i^2}]}{N_z}$$

10. High gray level emphasis (HGLE):

$$HGLE = \frac{\sum_{i=1}^{N_g} \sum_{j=1}^{N_d} i^2 \mathbf{P}(i,j)}{N_z}$$

11. Small dependence low gray level emphasis (SDLGLE):

$$SDLGLE = \frac{\sum_{i=1}^{N_g} \sum_{j=1}^{N_d} [\frac{p(i,j)}{i^2 j^2}]}{N_z}$$

12. Small dependence high gray level emphasis (SDHGLE):

$$SDHGLE = \frac{\sum_{i=1}^{N_g} \sum_{j=1}^{N_s} [\frac{p(i,j)i^2}{j^2}]}{N_z}$$

13. Large dependence low gray level emphasis (LDLGLE):

$$LDLGLE = \frac{\sum_{i=1}^{N_g} \sum_{j=1}^{N_d} [\frac{p(i,j)j^2}{i^2}]}{N_z}$$

14. Large dependence high gray level emphasis (LDHGLE):

$$LDHGLE = \frac{\sum_{i=1}^{N_g} \sum_{j=1}^{N_d} [p(i,j)j^2 i^2]}{N_z}$$

❖ **Wavelet features: Histogram-based and textural features of wavelet decompositions**

Undecimated Wavelet transformation was adopted to capture the features on the regions of interest from different scales in low- and high-frequencies. Here, we applied “coiflet1” wavelet transformation to decompose the original image into 4 parts. L and H indicated low- and high-

pass functions, respectively. The wavelet decompositions of the original image X were labeled as X_{LL}, X_{LH}, X_{HL}, and X_{HH}. For example, X_{LH} represents the image filtered with low-pass function in the x-directions and high-pass function in the y-direction, as follows:

$$X_{LH}(i,j) = \sum_{p=1}^{Nl} \sum_{q=1}^{Nh} L(p)H(q)X(i+p, j+q)$$

Where Nl is the length of filter L, Nh is the length of filter H. For each decomposition, we computed the histogram-based features and the textural features. In addition, features from wavelet decompositions X_{LLL} were also extracted.

▪ **LoG features: Histogram-based and textural features of Laplacian of Gaussian**

The Laplacian of an image brings out area of rapid intensity change and is usually used for edge detection. A Gaussian filter is applied prior to the Laplacian to smooth the image and reduce noise.

The equation of LoG with 2D kernel various

$$LoG(x,y) = -\frac{1}{\pi\sigma^4}\left[1 - \frac{x^2+y^2}{2\sigma^2}\right] e^{-\frac{x^2+y^2}{2\sigma^2}}$$

$$\sigma \, \epsilon [1mm, 3mm, 5mm]$$

Textural size (fine to coarse) was highlighted by modifying Gaussian radius parameter (from 0.5mm to 5mm, 0.5mm increment). Histogram-based and textural features of Laplacian of Gaussian were extracted.

▪ **gradient features: Histogram-based and textural features of gradient magnitude images**

An image gradient is a directional change in the intensity or color in an image. Mathematically, the gradient of the image intensity function at each image point is a 2D vector with the components given by the derivatives in the horizontal and vertical directions.

$$\nabla f = \begin{bmatrix} g_x \\ g_y \end{bmatrix} = \begin{bmatrix} \frac{\partial f}{\partial x} \\ \frac{\partial f}{\partial y} \end{bmatrix},$$

where:

$\frac{\partial f}{\partial x}$ is the derivative with respect to x (gradient in x direction)

$\frac{\partial f}{\partial y}$ is the derivative with respect to x (gradient in y direction).

The gradient magnitude is calculated by the following formula:

$$G = \sqrt{{g_x}^2 + {g_y}^2}.$$

Based on the calculated gradient magnitude G, histogram-based and textural features were extracted.

Supplementary Table 2. Comparison of patient and tumor characteristics between the training and validation cohort

Characteristics	Training cohort (n = 238)	Validation cohort (n = 119)	P value
Age at initial diagnosis (years)			0.159
≤40	68 (28.6)	25 (21.0)	
>40	170 (71.4)	94 (79.0)	
Sex			0.652
Male	133 (55.9)	63 (52.9)	
Female	105 (44.1)	56 (47.1)	
KPS score on admission			0.809
<80	73 (30.7)	38 (31.9)	
≥80	165 (69.3)	81 (68.1)	
Tumor location			0.466
Frontal lobe	88 (37.0)	51 (42.9)	
Temporal lobe	48 (20.2)	19 (16.0)	
Parietal lobe	10 (4.2)	8 (6.7)	
Occipital lobe	6 (2.5)	3 (2.5)	
Insular lobe	5 (2.1)	5 (4.2)	
Multicenter	81 (34.0)	33 (27.7)	
Laterality			0.646
Left	113 (47.5)	55 (46.2)	
Right	120 (50.4)	63 (52.9)	
Bilateral	5 (2.1)	1 (0.8)	
WHO grade			0.815

II	112 (47.1)	58 (48.7)	
III	53 (22.3)	23 (19.3)	
IV	73 (30.7)	38 (31.9)	
Histologic type			0.532
Astrocytoma	65 (27.3)	26 (21.8)	
Oligodendroglioma	101 (42.4)	55 (46.2)	
Glioblastoma	72 (30.3)	38 (31.9)	
Extent of surgical resection			0.087
Gross total resection	188 (79.0)	84 (70.6)	
Subtotal resection	50 (21.0)	35 (29.4)	
IDH mutation			0.260
Yes	105 (44.1)	60 (50.4)	
No	133 (55.9)	59 (49.6)	
1p/19q codeletion			0.612
Yes	61 (25.6)	34 (28.6)	
No	177 (74.4)	85 (71.4)	
TERT mutation			0.822
Yes	122 (51.3)	63 (52.9)	
No	116 (48.7)	56 (47.1)	
Radiotherapy			0.897
Yes	177 (74.4)	90 (75.6)	
No	61 (25.6)	29 (24.4)	
Chemotherapy			0.075
Temozolomide	53 (22.3)	32 (26.9)	
Nimustine / Fotemustine	93 (39.1)	32 (26.9)	
None	92 (39.1)	55 (46.2)	
Median PFS (months)	30.8	29.6	0.402
Median OS (months)	34.5	33.0	0.332

Note: Numbers in parenthesis are percentage; KPS, Karnofsky Performance Status; WHO, World Health Organization; *IDH*, isocitrate dehydrogenase; *TERT*, telomerase reverse transcriptase; PFS, progression free survival; OS, overall survival.

Supplementary Table 3: The number of retained features after each step of feature selection

	Selection	IDH	1p/19q	TERT
**T1WI**	Step 1	576	576	576
	Step 2	299	299	299
	Step 3	267	248	17
	Step 4	18	24	5
	Step 5	4	2	4
**T2WI**	Step 1	1134	1134	1134
	Step 2	673	673	673
	Step 3	631	518	32
	Step 4	39	27	10
	Step 5	7	3	3
**T2-FLAIR**	Step 1	840	840	840
	Step 2	544	544	773
	Step 3	526	480	9
	Step 4	33	16	6
	Step 5	7	3	2
**cT1WI**	Step 1	618	618	618
	Step 2	328	328	514
	Step 3	301	280	20
	Step 4	20	25	11
	Step 5	8	4	2
**ADC**	Step 1	832	832	832
	Step 2	584	584	584
	Step 3	548	489	10
	Step 4	23	24	5
	Step 5	6	5	3

Supplementary Table 4: The final features used for construction of single-layered radiomic signatures

**IDH mutation**	
**Sequences**	**Feature name**
T1WI	Tumor_original_shape_Maximum2DDiameterRow<br>Edema_original_shape_Maximum3DDiameter<br>Edema_wavelet-HH_gldm_LargeDependenceLowGrayLevelEmphasis<br>Edema_wavelet-LH_glszm_LargeAreaLowGrayLevelEmphasis
cT1WI	Tumor_log-sigma-1-0-mm-3D_firstorder_RootMeanSquared<br>Tumor_log-sigma-1-0-mm-3D_glrlm_GrayLevelNonUniformity<br>Tumor_log-sigma-3-0-mm-3D_glszm_GrayLevelNonUniformity<br>Tumor_log-sigma-5-0-mm-3D_glrlm_LongRunHighGrayLevelEmphasis

	Tumor_wavelet-LH_firstorder_Kurtosis<br>Edema_original_shape_Maximum3DDiameter<br>Edema_log-sigma-1-0-mm-3D_glszm_LargeAreaLowGrayLevelEmphasis<br>Edema_log-sigma-3-0-mm-3D_firstorder_Median
T2WI	Tumor_log-sigma-1-0-mm-3D_firstorder_Median<br>Tumor_wavelet-HL_gldm_GrayLevelNonUniformity<br>Tumor_wavelet-HL_firstorder_Kurtosis<br>Tumor_wavelet-LH_gldm_LargeDependenceHighGrayLevelEmphasis<br>original_firstorder_Kurtosis<br>Edema_original_shape_Maximum3DDiameter<br>Edema_gradient_firstorder_Kurtosis
T2-FLAIR	Tumor_log-sigma-3-0-mm-3D_glszm_GrayLevelNonUniformity<br>Tumor_wavelet-HL_glcm_ClusterShade<br>Tumor_wavelet-LL_firstorder_Kurtosis<br>Edema_original_shape_Maximum3DDiameter<br>Edema_wavelet-LL_gldm_LargeDependenceHighGrayLevelEmphasis<br>Edema_wavelet-LL_firstorder_Minimum<br>Edema_wavelet-LH_firstorder_Kurtosis
ADC	Tumor_original_shape_Maximum2DDiameterRow<br>Tumor_log-sigma-3-0-mm-3D_firstorder_Median<br>Tumor_log-sigma-5-0-mm-3D_firstorder_90Percentile<br>Tumor_original_firstorder_Kurtosis<br>Edema_original_shape_Maximum3DDiameter<br>Edema_log-sigma-3-0-mm-3D_glcm_ClusterShade
**1p/19q codeletion**	
**Sequences**	**Feature name**
T1WI	Edema_log-sigma-5-0-mm-3D_firstorder_RootMeanSquared<br>Edema_log-sigma-5-0-mm-3D_glszm_SmallAreaHighGrayLevelEmphasis
cT1WI	Tumor_log-sigma-1-0-mm-3D_firstorder_Mean<br>Tumor_original_gldm_LargeDependenceHighGrayLevelEmphasis<br>Tumor_original_firstorder_Kurtosis<br>Edema_log-sigma-5-0-mm-3D_firstorder_RootMeanSquared
T2WI	Edema_gradient_firstorder_Kurtosis<br>Edema_wavelet-HL_gldm_LargeDependenceEmphasis<br>Edema_wavelet-HH_firstorder_Kurtosis
T2-FLAIR	Tumor_log-sigma-3-0-mm-3D_glcm_ClusterShade<br>Tumor_wavelet-HL_glcm_ClusterShade<br>Edema_log-sigma-1-0-mm-3D_firstorder_RootMeanSquared
ADC	Tumor_log-sigma-5-0-mm-3D_firstorder_90Percentile<br>Edema_log-sigma-3-0-mm-3D_gldm_GrayLevelNonUniformity<br>Edema_wavelet-HL_firstorder_Kurtosis<br>Edema_wavelet-LH_glrlm_LongRunHighGrayLevelEmphasis<br>Edema_original_glszm_GrayLevelVariance

**TERT promoter mutation**	
T1WI	Tumor_log-sigma-5-0-mm-3D_firstorder_10Percentile<br>Tumor_wavelet-LL_glcm_JointAverage<br>Tumor_wavelet-LL_glcm_ClusterShade<br>Tumor_original_firstorder_90Percentile
cT1WI	Tumor_wavelet-LL_firstorder_Skewness<br>Tumor_original_glcm_Correlation
T2WI	Tumor_gradient_glszm_LargeAreaHighGrayLevelEmphasis<br>Tumor_log-sigma-1-0-mm-3D_firstorder_Median<br>Tumor_original_firstorder_Kurtosis
T2-FLAIR	Tumor_original_shape_Sphericity<br>Tumor_wavelet-LL_firstorder_Minimum
ADC	Tumor_log-sigma-1-0-mm-3D_firstorder_Median<br>Tumor_wavelet-HL_glcm_ClusterShade<br>Tumor_original_firstorder_Minimum

Supplementary Table 5. The performance of single-layered radiomic signatures for prediction of IDH mutation status

Sequence	WHO grade	Training cohort						Validation cohort					
		AUC	ACC	SEN	SPE	PPV	NPV	AUC	ACC	SEN	SPE	PPV	NPV
T1WI	II-IV	0.838 (0.791-0.880)	0.798 (0.756-0.840)	0.857 (0.798-0.909)	0.752 (0.687-0.813)	0.732 (0.661-0.795)	0.870 (0.818-0.917)	0.854 (0.792-0.911)	0.824 (0.765-0.882)	0.783 (0.694-0.868)	0.864 (0.786-0.933)	0.855 (0.776-0.926)	0.797 (0.717-0.877)
	II/III	0.785 (0.720-0.846)	0.758 (0.709-0.812)	0.889 (0.835-0.943)	0.561 (0.458-0.661)	0.752 (0.688-0.817)	0.771 (0.660-0.875)	0.868 (0.789-0.935)	0.840 (0.765-0.901)	0.837 (0.739-0.925)	0.842 (0.732-0.933)	0.857 (0.766-0.938)	0.821 (0.710-0.917)
	IV	0.542 (0.421-0.667)	0.890 (0.795-0.904)	0.333 (0.200-0.500)	0.940 (0.891-0.984)	0.333 (0.286-0.833)	0.940 (0.833-0.925)	0.818 (0.713-0.911)	0.789 (0.632-0.789)	0.647 (0.545-0.731)	0.905 (0.750-1.000)	0.846 (0.842-1.000)	0.760 (0.389-0.652)
cT1WI	II-IV	0.906 (0.874-0.939)	0.861 (0.828-0.899)	0.857 (0.800-0.911)	0.865 (0.815-0.915)	0.833 (0.774-0.892)	0.885 (0.837-0.929)	0.869 (0.807-0.927)	0.815 (0.756-0.874)	0.767 (0.677-0.855)	0.864 (0.789-0.931)	0.852 (0.765-0.925)	0.785 (0.700-0.867)
	II/III	0.838 (0.779-0.893)	0.830 (0.782-0.879)	0.879 (0.824-0.930)	0.758 (0.672-0.845)	0.845 (0.786-0.904)	0.806 (0.724-0.880)	0.882 (0.803-0.948)	0.852 (0.790-0.914)	0.837 (0.738-0.923)	0.868 (0.771-0.951)	0.878 (0.784-0.956)	0.825 (0.722-0.919)
	IV	0.935 (0.893-0.969)	0.932 (0.836-0.932)	0.500 (0.333-0.667)	0.970 (0.932-1.000)	0.600 (0.500-1.000)	0.956 (0.866-0.952)	0.846 (0.704-0.975)	0.737 (0.579-0.763)	0.588 (0.484-0.682)	0.857 (0.667-1.000)	0.769 (0.786-1.000)	0.720 (0.333-0.609)
T2WI	II-IV	0.875 (0.836-0.912)	0.832 (0.790-0.870)	0.829 (0.764-0.890)	0.835 (0.781-0.885)	0.798 (0.735-0.858)	0.860 (0.807-0.910)	0.817 (0.747-0.884)	0.782 (0.714-0.840)	0.700 (0.603-0.797)	0.864 (0.782-0.932)	0.840 (0.745-0.915)	0.739 (0.645-0.825)
	II/III	0.836 (0.780-0.890)	0.812 (0.764-0.861)	0.859 (0.800-0.915)	0.742 (0.657-0.833)	0.833 (0.770-0.893)	0.778 (0.697-0.864)	0.822 (0.730-0.907)	0.802 (0.728-0.877)	0.744 (0.630-0.846)	0.868 (0.775-0.953)	0.865 (0.769-0.952)	0.750 (0.641-0.857)
	IV	0.697 (0.584-0.803)	0.877 (0.767-0.890)	0.333 (0.182-0.500)	0.925 (0.869-0.969)	0.286 (0.250-0.750)	0.939 (0.831-0.924)	0.804 (0.687-0.900)	0.737 (0.579-0.763)	0.588 (0.484-0.680)	0.857 (0.692-1.000)	0.769 (0.789-1.000)	0.720 (0.333-0.609)
T2-FLAIR	II-IV	0.879 (0.840-0.916)	0.811 (0.769-0.857)	0.848 (0.789-0.906)	0.782 (0.719-0.844)	0.754 (0.686-0.821)	0.867 (0.817-0.918)	0.846 (0.788-0.906)	0.798 (0.739-0.857)	0.750 (0.662-0.839)	0.847 (0.774-0.922)	0.833 (0.755-0.917)	0.769 (0.681-0.850)
	II/III	0.837 (0.785-0.892)	0.776 (0.727-0.830)	0.869 (0.811-0.926)	0.636 (0.543-0.735)	0.782 (0.718-0.845)	0.764 (0.667-0.862)	0.879 (0.814-0.940)	0.802 (0.728-0.877)	0.791 (0.689-0.886)	0.816 (0.710-0.917)	0.829 (0.722-0.923)	0.775 (0.667-0.881)
	IV	0.756 (0.648-0.851)	0.890 (0.795-0.918)	0.500 (0.333-0.667)	0.925 (0.862-0.969)	0.375 (0.357-0.800)	0.954 (0.862-0.950)	0.737 (0.636-0.830)	0.789 (0.658-0.816)	0.647 (0.556-0.739)	0.905 (0.750-1.000)	0.846 (0.850-1.000)	0.760 (0.368-0.667)
ADC	II-IV	0.866 (0.824-0.905)	0.819 (0.777-0.861)	0.876 (0.819-0.925)	0.774 (0.715-0.836)	0.754 (0.692-0.819)	0.888 (0.836-0.933)	0.842 (0.775-0.902)	0.807 (0.748-0.866)	0.783 (0.685-0.868)	0.831 (0.746-0.906)	0.825 (0.729-0.906)	0.790 (0.696-0.873)
	II/III	0.801 (0.732-0.865)	0.776 (0.721-0.836)	0.899 (0.850-0.944)	0.591 (0.484-0.696)	0.767 (0.704-0.833)	0.796 (0.692-0.886)	0.859 (0.786-0.924)	0.815 (0.741-0.877)	0.814 (0.711-0.907)	0.816 (0.714-0.914)	0.833 (0.737-0.927)	0.795 (0.676-0.895)
	IV	0.706 (0.594-0.814)	0.918 (0.836-0.932)	0.500 (0.333-0.667)	0.955 (0.908-1.000)	0.500 (0.455-1.000)	0.955 (0.866-0.952)	0.787 (0.691-0.862)	0.789 (0.658-0.816)	0.706 (0.621-0.786)	0.857 (0.667-1.000)	0.800 (0.810-1.000)	0.783 (0.400-0.696)

Note: AUC = area under the curve; SEN = sensitivity; SPE = specificity; ACC = accuracy; PPV = positive predictive value; NPV = negative predictive value; Data within parentheses represent 95% confidence interval (CI).

Supplementary Table 6. The performance of single-layered radiomic signatures for prediction of 1p/19q codeletion status

Sequence	WHO grade	Training cohort						Validation cohort					
		AUC	ACC	SEN	SPE	PPV	NPV	AUC	ACC	SEN	SPE	PPV	NPV
T1WI	II-IV	0.763 (0.723-0.802)	0.676 (0.626-0.727)	0.902 (0.841-0.962)	0.599 (0.535-0.659)	0.437 (0.368-0.508)	0.946 (0.912-0.981)	0.722 (0.639-0.805)	0.697 (0.630-0.765)	0.794 (0.667-0.909)	0.659 (0.581-0.744)	0.482 (0.373-0.596)	0.889 (0.817-0.954)
	II/III	0.650 (0.600-0.704)	0.576 (0.509-0.642)	0.902 (0.833-0.966)	0.385 (0.308-0.466)	0.462 (0.385-0.539)	0.870 (0.784-0.952)	0.594 (0.505-0.682)	0.568 (0.469-0.654)	0.794 (0.677-0.907)	0.404 (0.286-0.526)	0.491 (0.378-0.597)	0.731 (0.583-0.870)
cT1WI	II-IV	0.794 (0.745-0.844)	0.706 (0.660-0.756)	0.918 (0.852-0.972)	0.633 (0.576-0.694)	0.463 (0.387-0.539)	0.957 (0.925-0.984)	0.815 (0.751-0.878)	0.723 (0.655-0.790)	0.794 (0.677-0.897)	0.694 (0.609-0.774)	0.509 (0.391-0.620)	0.894 (0.831-0.953)
	II/III	0.703 (0.636-0.770)	0.612 (0.552-0.673)	0.918 (0.860-0.969)	0.433 (0.357-0.515)	0.487 (0.412-0.568)	0.900 (0.827-0.962)	0.695 (0.599-0.787)	0.605 (0.519-0.691)	0.794 (0.667-0.906)	0.468 (0.356-0.600)	0.519 (0.404-0.633)	0.759 (0.630-0.889)
T2WI	II-IV	0.767 (0.728-0.804)	0.668 (0.618-0.718)	0.951 (0.898-0.985)	0.571 (0.511-0.633)	0.433 (0.360-0.500)	0.971 (0.942-0.991)	0.733 (0.661-0.797)	0.630 (0.563-0.706)	0.794 (0.676-0.897)	0.565 (0.481-0.655)	0.422 (0.323-0.532)	0.873 (0.796-0.935)
	II/III	0.655 (0.607-0.703)	0.588 (0.527-0.655)	0.951 (0.902-1.000)	0.375 (0.300-0.454)	0.472 (0.400-0.547)	0.929 (0.857-1.000)	0.600 (0.516-0.684)	0.519 (0.420-0.605)	0.794 (0.675-0.903)	0.319 (0.213-0.435)	0.458 (0.350-0.557)	0.682 (0.500-0.846)
T2-FLAIR	II-IV	0.818 (0.765-0.865)	0.773 (0.727-0.815)	0.820 (0.736-0.891)	0.757 (0.703-0.804)	0.538 (0.457-0.624)	0.924 (0.887-0.956)	0.758 (0.662-0.843)	0.748 (0.681-0.815)	0.588 (0.444-0.731)	0.812 (0.736-0.875)	0.556 (0.406-0.692)	0.831 (0.762-0.896)
	II/III	0.745 (0.677-0.808)	0.691 (0.636-0.745)	0.820 (0.737-0.897)	0.615 (0.542-0.693)	0.556 (0.477-0.636)	0.853 (0.780-0.915)	0.655 (0.546-0.760)	0.630 (0.543-0.716)	0.588 (0.444-0.730)	0.660 (0.540-0.771)	0.556 (0.412-0.692)	0.689 (0.574-0.804)
ADC	II-IV	0.790 (0.740-0.836)	0.752 (0.706-0.798)	0.836 (0.754-0.909)	0.723 (0.663-0.778)	0.510 (0.424-0.593)	0.928 (0.886-0.964)	0.674 (0.574-0.767)	0.697 (0.622-0.765)	0.676 (0.536-0.800)	0.706 (0.624-0.787)	0.479 (0.349-0.600)	0.845 (0.771-0.912)
	II/III	0.683 (0.605-0.750)	0.673 (0.606-0.727)	0.836 (0.750-0.914)	0.577 (0.495-0.657)	0.537 (0.452-0.624)	0.857 (0.783-0.923)	0.538 (0.424-0.647)	0.556 (0.457-0.642)	0.676 (0.533-0.808)	0.468 (0.348-0.588)	0.479 (0.357-0.600)	0.667 (0.531-0.800)

Note: AUC = area under the curve; SEN = sensitivity; SPE = specificity; ACC = accuracy; PPV = positive predictive value; NPV = negative predictive value; Data within parentheses represent 95% confidence interval (CI); the performance metrics of single-layered radiomic signatures in prediction of 1p/19q codeletion status in grade IV gliomas could not be calculated because no patients had 1p/19q codeletion.

Supplementary Table 7. The performance of single-layered radiomic signatures for prediction of TERT promoter mutation status

Sequence	WHO grade	Training cohort						Validation cohort					
		AUC	ACC	SEN	SPE	PPV	NPV	AUC	ACC	SEN	SPE	PPV	NPV
T1WI	II-IV	0.665 (0.603-0.726)	0.639 (0.588-0.689)	0.516 (0.433-0.590)	0.767 (0.703-0.835)	0.700 (0.618-0.779)	0.601 (0.534-0.664)	0.535 (0.445-0.629)	0.563 (0.487-0.639)	0.429 (0.328-0.536)	0.714 (0.612-0.810)	0.628 (0.500-0.750)	0.526 (0.429-0.619)
	II/III	0.705 (0.638-0.770)	0.661 (0.600-0.721)	0.562 (0.471-0.653)	0.753 (0.671-0.826)	0.682 (0.590-0.769)	0.646 (0.570-0.721)	0.487 (0.378-0.598)	0.519 (0.432-0.617)	0.378 (0.261-0.500)	0.694 (0.568-0.810)	0.607 (0.444-0.750)	0.472 (0.368-0.587)
	IV	0.569 (0.464-0.686)	0.589 (0.493-0.685)	0.429 (0.310-0.550)	0.806 (0.677-0.920)	0.750 (0.600-0.889)	0.510 (0.391-0.625)	0.633 (0.479-0.778)	0.658 (0.553-0.789)	0.556 (0.364-0.737)	0.750 (0.579-0.900)	0..667 (0.455-0.867)	0.652 (0.500-0.808)
cT1WI	II-IV	0.661 (0.596-0.713)	0.651 (0.597-0.702)	0.648 (0.576-0.723)	0.655 (0.575-0.726)	0.664 (0.585-0.736)	0.639 (0.563-0.709)	0.699 (0.619-0.775)	0.647 (0.571-0.723)	0.698 (0.597-0.789)	0.589 (0.480-0.700)	0.657 (0.559-0.750)	0.635 (0.519-0.741)
	II/III	0.649 (0.573-0.717)	0.630 (0.564-0.691)	0.538 (0.436-0.629)	0.718 (0.639-0.800)	0.642 (0.533-0.743)	0.622 (0.544-0.698)	0.675 (0.569-0.765)	0.617 (0.531-0.704)	0.667 (0.545-0.787)	0.556 (0.417-0.689)	0.652 (0.533-0.762)	0.571 (0.429-0.719)
	IV	0.619 (0.505-0.737)	0.699 (0.616-0.781)	0.857 (0.771-0.939)	0.484 (0.333-0.630)	0.692 (0.588-0.796)	0.714 (0.545-0.870)	0.758 (0.625-0.886)	0.711 (0.579-0.816)	0.778 (0.611-0.938)	0.650 (0.471-0.826)	0.667 (0.500-0.840)	0.765 (0.588-0.933)
T2WI	II-IV	0.650 (0.587-0.710)	0.660 (0.609-0.710)	0.746 (0.685-0.811)	0.569 (0.495-0.645)	0.645 (0.584-0.710)	0.680 (0.604-0.761)	0.573 (0.481-0.657)	0.529 (0.454-0.605)	0.571 (0.467-0.679)	0.482 (0.367-0.596)	0.554 (0.452-0.655)	0.500 (0.383-0.615)
	II/III	0.670 (0.597-0.738)	0.655 (0.594-0.715)	0.650 (0.565-0.740)	0.659 (0.571-0.742)	0.642 (0.557-0.730)	0.667 (0.583-0.750)	0.554 (0.441-0.662)	0.506 (0.420-0.593)	0.556 (0.431-0.673)	0.444 (0.313-0.586)	0.556 (0.429-0.679)	0.444 (0.310-0.579)
	IV	0.560 (0.436-0.678)	0.671 (0.589-0.753)	0.929 (0.857-0.979)	0.323 (0.182-0.469)	0.650 (0.544-0.754)	0.769 (0.563-0.933)	0.606 (0.441-0.759)	0.579 (0.447-0.711)	0.611 (0.409-0.789)	0.550 (0.364-0.737)	0.550 (0.353-0.733)	0.611 (0.412-0.800)
T2-FLAIR	II-IV	0.582 (0.521-0.646)	0.580 (0.529-0.634)	0.484 (0.412-0.561)	0.681 (0.614-0.754)	0.615 (0.536-0.698)	0.556 (0.490-0.625)	0.482 (0.396-0.574)	0.496 (0.420-0.580)	0.397 (0.302-0.500)	0.607 (0.508-0.712)	0.532 (0.410-0.660)	0.472 (0.379-0.571)
	II/III	0.617 (0.544-0.688)	0.636 (0.576-0.697)	0.575 (0.479-0.667)	0.694 (0.615-0.776)	0.639 (0.544-0.726)	0.634 (0.545-0.716)	0.419 (0.316-0.533)	0.444 (0.358-0.531)	0.311 (0.200-0.436)	0.611 (0.471-0.750)	0.500 (0.343-0.647)	0.415 (0.296-0.526)
	IV	0.520 (0.406-0.645)	0.452 (0.356-0.548)	0.310 (0.200-0.425)	0.645 (0.500-0.793)	0.542 (0.385-0.714)	0.408 (0.298-0.521)	0.592 (0.428-0.756)	0.605 (0.474-0.737)	0.611 (0.400-0.810)	0.600 (0.400-0.778)	0.579 (0.381-0.762)	0.632 (0.444-0.813)
ADC	II-IV	0.634 (0.575-0.696)	0.660 (0.605-0.710)	0.754 (0.692-0.814)	0.560 (0.480-0.638)	0.643 (0.582-0.712)	0.684 (0.600-0.759)	0.603 (0.520-0.686)	0.605 (0.529-0.672)	0.730 (0.642-0.825)	0.464 (0.360-0.578)	0.605 (0.507-0.693)	0.605 (0.490-0.738)
	II/III	0.665 (0.592-0.733)	0.667 (0.606-0.727)	0.663 (0.575-0.753)	0.671 (0.585-0.753)	0.654 (0.564-0.736)	0.679 (0.597-0.763)	0.526 (0.424-0.635)	0.531 (0.444-0.630)	0.644 (0.529-0.769)	0.389 (0.268-0.529)	0.569 (0.453-0.685)	0.467 (0.333-0.619)
	IV	0.520 (0.402-0.636)	0.644 (0.548-0.740)	0.929 (0.857-0.978)	0.258 (0.133-0.394)	0.629 (0.524-0.732)	0.727 (0.500-0.917)	0.781 (0.645-0.891)	0.763 (0.658-0.868)	0.944 (0.833-1.000)	0.600 (0.429-0.778)	0.680 (0.524-0.821)	0.923 (0.789-1.000)

Note: AUC = area under the curve; SEN = sensitivity; SPE = specificity; ACC = accuracy; PPV = positive predictive value; NPV = negative predictive value; Data within parentheses represent 95% confidence interval (CI).

Supplementary Note 2: The hyper-parameters setting of Bayesian-regularization neural networks (BRNN) and the results of 10-fold cross-validation

10-fold cross-validation was used to identify the optimal hyper-parameters in BRNN (epoch, neuron, mu, and defaults for others).

epoch: maximum number of epochs(iterations) to train

neuron: ndicates the number of neurons

mu: controls the behaviour of the Gauss-Newton optimization algorithm

IDH mutation status:

T1WI

AUC values in 10 folds

best_auc (0.818), best_auc_cv1 (0.831), best_auc_cv2 (0.909), best_auc_cv3 (0.783), best_auc_cv4 (0.762), best_auc_cv5 (0.754), best_auc_cv6 (0.831), best_auc_cv7 (0.862), best_auc_cv8 (0.838), best_auc_cv9 (0.890), best_auc_cv10 (0.721)

hyper-parameters: best_epoch (300), best_neuron (2), best_mu (0.005)

c**T1WI**

AUC values in 10 folds

best_auc (0.874), best_auc_cv1 (0.785), best_auc_cv2 (0.838), best_auc_cv3 (0.931), best_auc_cv4 (0.944), best_auc_cv5 (0.838), best_auc_cv6 (0.909), best_auc_cv7 (0.839), best_auc_cv8 (0.916), best_auc_cv9 (0.793), best_auc_cv10 (0.942)

hyper-parameters: best_epoch (100), best_neuron (1), best_mu (0.001)

T2WI

AUC values in 10 folds

best_auc (0.865), best_auc_cv1 (0.951), best_auc_cv2 (0.664), best_auc_cv3 (0.909), best_auc_cv4 (0.779), best_auc_cv5 (0.854), best_auc_cv6 (0.877), best_auc_cv7 (0.943), best_auc_cv8 (0.846), best_auc_cv9 (0.851), best_auc_cv10 (0.977)

hyper-parameters: best_epoch (100), best_neuron (3), best_mu (0.002)

T2-FLAIR

AUC values in 10 folds

best_auc (0.872), best_auc_cv1 (1.000), best_auc_cv2 (0.903), best_auc_cv3 (0.814),

best_auc_cv4 (0.909), best_auc_cv5 (0.832), best_auc_cv6 (0.881), best_auc_cv7 (0.908), best_auc_cv8 (0.929), best_auc_cv9 (0.877), best_auc_cv10 (0.671)

hyper-parameters: best_epoch (250), best_neuron (2), best_mu (0.007)

ADC

AUC values in 10 folds

best_auc (0.849), best_auc_cv1 (0.790), best_auc_cv2 (0.857), best_auc_cv3 (0.831), best_auc_cv4 (0.762), best_auc_cv5 (1.000), best_auc_cv6 (0.684), best_auc_cv7 (0.874), best_auc_cv8 (0.860), best_auc_cv9 (0.857), best_auc_cv10 (0.977)

hyper-parameters: best_epoch (400), best_neuron (4), best_mu (0.007)

1p/19q codeletion status:

T1WI

AUC values in 10 folds

best_auc (0.764), best_auc_cv1 (0.824), best_auc_cv2 (0.806), best_auc_cv3 (0.764), best_auc_cv4 (0.824), best_auc_cv5 (0.882), best_auc_cv6 (0.861), best_auc_cv7 (0.635), best_auc_cv8 (0.667), best_auc_cv9 (0.676), best_auc_cv10 (0.699)

hyper-parameters: best_epoch (400), best_neuron (2), best_mu (0.005)

c**T1WI**

AUC values in 10 folds

best_auc (0.790), best_auc_cv1 (0.759), best_auc_cv2 (0.750), best_auc_cv3 (0.861), best_auc_cv4 (0.815), best_auc_cv5 (0.849), best_auc_cv6 (0.917), best_auc_cv7 (0.892), best_auc_cv8 (0.725), best_auc_cv9 (0.618), best_auc_cv10 (0.713)

hyper-parameters: best_epoch (100), best_neuron (1), best_mu (0.001)

T2WI

AUC values in 10 folds

best_auc (0.763), best_auc_cv1 (0.794), best_auc_cv2 (0.750), best_auc_cv3 (0.806), best_auc_cv4 (0.639), best_auc_cv5 (0.778), best_auc_cv6 (0.784), best_auc_cv7 (0.778), best_auc_cv8 (0.716), best_auc_cv9 (0.861), best_auc_cv10 (0.722)

hyper-parameters: best_epoch (100), best_neuron (2), best_mu (0.001)

T2-FLAIR

AUC values in 10 folds

best_auc (0.815), best_auc_cv1 (0.951), best_auc_cv2 (0.814), best_auc_cv3 (0.873), best_auc_cv4 (0.889), best_auc_cv5 (0.769), best_auc_cv6 (0.741), best_auc_cv7 (0.620), best_auc_cv8 (0.676), best_auc_cv9 (0.926), best_auc_cv10 (0.889)

hyper-parameters: best_epoch (100), best_neuron (1), best_mu (0.001)

ADC

AUC values in 10 folds

best_auc (0.791), best_auc_cv1 (0.657), best_auc_cv2 (0.714), best_auc_cv3 (0.778), best_auc_cv4 (0.750), best_auc_cv5 (0.873), best_auc_cv6 (0.796), best_auc_cv7 (0.824), best_auc_cv8 (0.794), best_auc_cv9 (0.778), best_auc_cv10 (0.944)

hyper-parameters: best_epoch (100), best_neuron (5), best_mu (0.002)

TERT promoter mutation status:

T1WI

AUC values in 10 folds

best_auc (0.678), best_auc_cv1 (0.545), best_auc_cv2 (0.688), best_auc_cv3 (0.576), best_auc_cv4 (0.818), best_auc_cv5 (0.692), best_auc_cv6 (0.646), best_auc_cv7 (0.771), best_auc_cv8 (0.523), best_auc_cv9 (0.611), best_auc_cv10 (0.911)

hyper-parameters: best_epoch (50), best_neuron (1), best_mu (0.001)

c**T1WI**

AUC values in 10 folds

best_auc (0.657), best_auc_cv1 (0.764), best_auc_cv2 (0.591), best_auc_cv3 (0.788), best_auc_cv4 (0.596), best_auc_cv5 (0.561), best_auc_cv6 (0.597), best_auc_cv7 (0.729), best_auc_cv8 (0.621), best_auc_cv9 (0.660), best_auc_cv10 (0.667)

hyper-parameters: best_epoch (700), best_neuron (1), best_mu (0.002)

T2WI

AUC values in 10 folds

best_auc (0.634), best_auc_cv1 (0.653), best_auc_cv2 (0.778), best_auc_cv3 (0.632), best_auc_cv4 (0.506), best_auc_cv5 (0.558), best_auc_cv6 (0.667), best_auc_cv7 (0.625), best_auc_cv8 (0.742), best_auc_cv9 (0.682), best_auc_cv10 (0.500)

hyper-parameters: best_epoch (100), best_neuron (1), best_mu (0.004)

T2-FLAIR

AUC values in 10 folds

best_auc (0.609), best_auc_cv1 (0.636), best_auc_cv2 (0.590), best_auc_cv3 (0.528), best_auc_cv4 (0.727), best_auc_cv5 (0.618), best_auc_cv6 (0.494), best_auc_cv7 (0.508), best_auc_cv8 (0.674), best_auc_cv9 (0.681), best_auc_cv10 (0.632)

hyper-parameters: best_epoch (100), best_neuron (1), best_mu (0.001)

ADC

AUC values in 10 folds

best_auc (0.602), best_auc_cv1 (0.576), best_auc_cv2 (0.778), best_auc_cv3 (0.523), best_auc_cv4 (0.625), best_auc_cv5 (0.629), best_auc_cv6 (0.420), best_auc_cv7 (0.444), best_auc_cv8 (0.736), best_auc_cv9 (0.688), best_auc_cv10 (0.601)

hyper-parameters: best_epoch (100), best_neuron (1), best_mu (0.001)

Supplementary Note 3: Confusion matrix of the image fusion models for predicting IDH, 1p/19q, and TERT status

IDH mutation status:

0-wild type; 1-mutation

[[actual 0, predict 0], [actual 0, predict 1]

[actual 1, predict 0], [actual 1, predict 1]]

Confusion matrix in the training cohort:

[[113 20]

[11 94]]

Confusion matrix in the validation cohort:

[[53 6]

[15 45]]

1p/19q codeletion status:

0- intact; 1-codeletion

[[actual 0, predict 0], [actual 0, predict 1]

[actual 1, predict 0], [actual 1, predict 1]]

Confusion matrix in the training cohort:

[[112 65]

[5 56]]

Confusion matrix in the validation cohort:

[[59 26]

[7 27]]

TERT promoter mutation status:

0-wild type; 1-mutation

[[actual 0, predict 0], [actual 0, predict 1]

[actual 1, predict 0], [actual 1, predict 1]]

Confusion matrix in the training cohort:

[[59 57]

[20 102]]

Confusion matrix in the validation cohort:

[[25 31]

[10 53]]

Supplementary Note 4: The formula of *image fusion model*s for predicting IDH mutation, 1p19q codeletion, and TERT promoter mutation, which were built by logistic regression based on the scores of single-layered methods

Image fusion model for predicting IDH mutation

-0.0491 + 0.3785*cT1WI + 0.7432*ADC

Image fusion model for predicting 1p19q codeletion

0.0002 + 1.0221* cT1WI

Image fusion model for predicting TERT promoter mutation

-0.2471 + 0.7025*cT1WI + 0.7925*ADC

Supplementary Figure 3: The barplots for the training cohort and the validation cohort with the prediction value for each patient: (a-b) IDH prediction values in the training cohort and validation cohort; (c-d) 1p/19q prediction values in the training and validation cohorts; and (e-f) TERT prediction values in the training and validation cohorts.

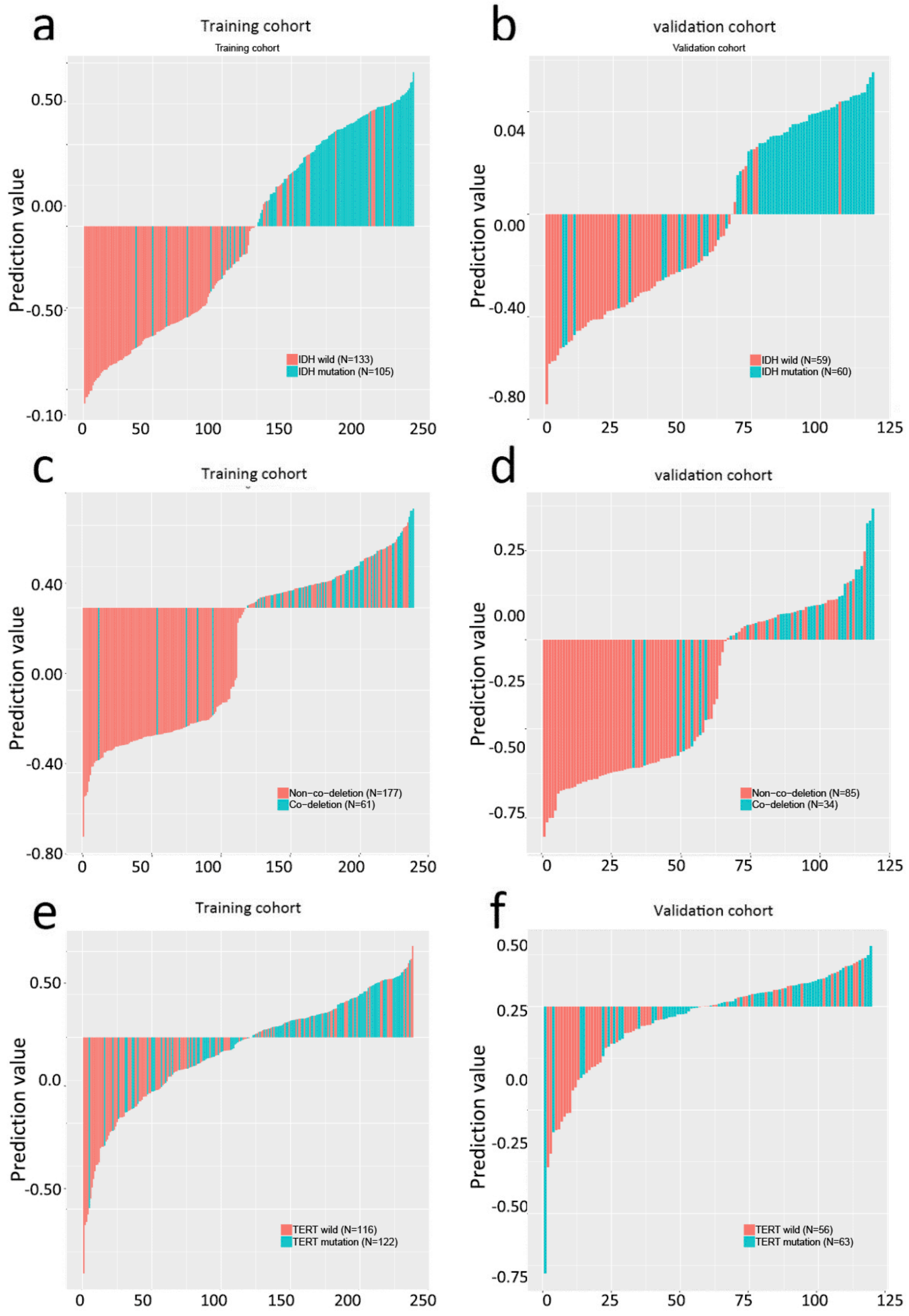

Supplementary Figure 4. Kaplan-Meier curves of molecular groups predicted by image fusion models. (a) PFS in the training cohort; (b) PFS in the validation cohort; (c) OS in the training cohort; and (d) OS in the validation cohort.

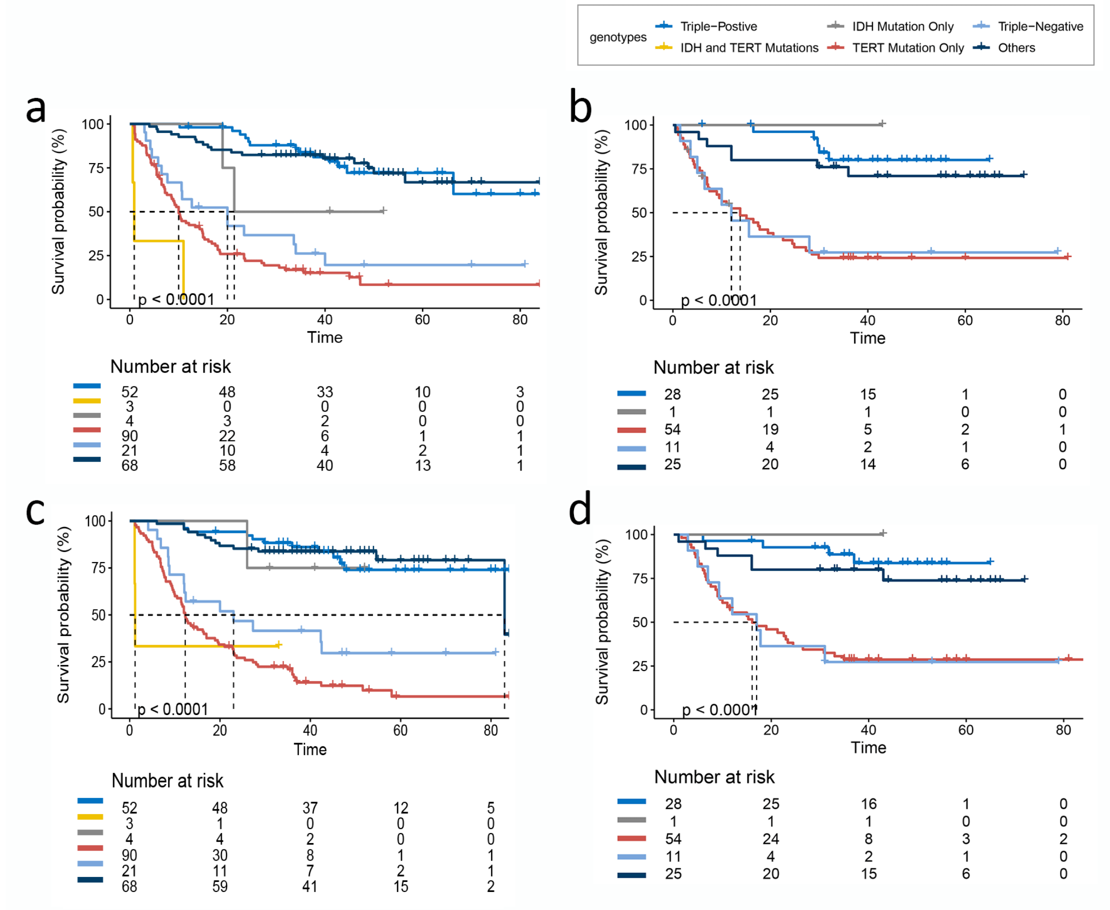

Supplementary Note 5: The HRs and 95%CI for WHO grade and the predictive molecular groups in assessment of PFS and OS

PFS prediction:

```
                            coef exp(coef) se(coef)      z Pr(>|z|)    
as.factor(pred.class)2  3.12543  22.76978  0.65919  4.741 2.12e-06 ***
as.factor(pred.class)3  0.52551   1.69132  0.76330  0.688 0.491160    
as.factor(pred.class)4  1.22907   3.41803  0.36078  3.407 0.000658 ***
as.factor(pred.class)5  1.11413   3.04691  0.39814  2.798 0.005136 ** 
as.factor(pred.class)6 -0.01607   0.98406  0.36910 -0.044 0.965276    
WHO.Grade               0.85308   2.34687  0.15352  5.557 2.75e-08 ***
---
Signif. codes:  0 ‘***’ 0.001 ‘**’ 0.01 ‘*’ 0.05 ‘.’ 0.1 ‘ ’ 1

                       exp(coef) exp(-coef) lower .95 upper .95
as.factor(pred.class)2   22.7698    0.04392    6.2554    82.882
as.factor(pred.class)3    1.6913    0.59126    0.3789     7.550
as.factor(pred.class)4    3.4180    0.29257    1.6853     6.932
as.factor(pred.class)5    3.0469    0.32820    1.3963     6.649
as.factor(pred.class)6    0.9841    1.01620    0.4774     2.029
WHO.Grade                 2.3469    0.42610    1.7371     3.171

Concordance= 0.799  (se = 0.017 )
Likelihood ratio test= 152.3  on 6 df,   p=<2e-16
Wald test            = 128.3  on 6 df,   p=<2e-16
Score (logrank) test = 179.3  on 6 df,   p=<2e-16
```

Note: Pred.class 2 = IDH and TERT mutation, Pred.class 3 = IDH mutation only, Pred.class 4 = TERT mutation only, Pred.class 5 = Triple negative, Pred-class 6 = others

OS prediction:

```
                             coef exp(coef) se(coef)      z Pr(>|z|)    
as.factor(pred.class)2  1.94157   6.96969  0.77848  2.494  0.01263 *  
as.factor(pred.class)3 -0.05871   0.94298  1.04803 -0.056  0.95532    
as.factor(pred.class)4  1.17227   3.22933  0.38348  3.057  0.00224 ** 
as.factor(pred.class)5  0.92955   2.53336  0.42867  2.168  0.03012 *  
as.factor(pred.class)6 -0.09292   0.91126  0.41065 -0.226  0.82098    
WHO.Grade               0.97421   2.64907  0.16466  5.916 3.29e-09 ***
---
Signif. codes:  0 ‘***’ 0.001 ‘**’ 0.01 ‘*’ 0.05 ‘.’ 0.1 ‘ ’ 1

                       exp(coef) exp(-coef) lower .95 upper .95
as.factor(pred.class)2    6.9697     0.1435    1.5156    32.052
as.factor(pred.class)3    0.9430     1.0605    0.1209     7.355
as.factor(pred.class)4    3.2293     0.3097    1.5230     6.847
as.factor(pred.class)5    2.5334     0.3947    1.0935     5.869
as.factor(pred.class)6    0.9113     1.0974    0.4075     2.038
WHO.Grade                 2.6491     0.3775    1.9184     3.658

Concordance= 0.806  (se = 0.017 )
Likelihood ratio test= 160.1  on 6 df,   p=<2e-16
Wald test            = 124  on 6 df,   p=<2e-16
Score (logrank) test = 179.7  on 6 df,   p=<2e-16
```

Note: Pred.class 2 = IDH and TERT mutation, Pred.class 3 = IDH mutation only, Pred.class 4 = TERT mutation only, Pred.class 5 = Triple negative, Pred-class 6 = others
